# Video Streaming or Telephone Communication During Emergency Medical Services Dispatch Calls: A Cluster Randomized Clinical Trial

**DOI:** 10.1001/jamanetworkopen.2025.19020

**Published:** 2025-07-01

**Authors:** Martin Faurholdt Gude, Jan Brink Valentin, Milena Meisner-Jensen, Natascha Hougaard Bohnstedt-Pedersen, Anne Kathrine Dalgaard, Ulla Væggemose, Rolf Ankerlund Blauenfeldt

**Affiliations:** 1 Department of Research & Development, Prehospital Emergency Medical Services, Central Denmark Region, Denmark; 2 Department of Clinical Medicine, Aarhus University, Denmark; 3 Danish Center for Health Services Research, Department of Clinical Medicine, Aalborg University, Aalborg, Denmark; 4 Department of Neurology & Danish Stroke Center, Aarhus University Hospital, Aarhus, Denmark

## Abstract

**Importance:**

Video streaming during emergency medical calls may enhance triage precision and optimize health resource allocation. However, its clinical safety and effect on key outcomes remain unexplored in randomized clinical trials.

**Objective:**

To assess whether video streaming during emergency calls reduces highest-urgency ambulance dispatches while ensuring safety through outcomes such as 24-hour hospital admissions among initially nonconveyed patients, mortality, and intensive care unit (ICU) admissions.

**Design, Setting, and Participants:**

This cluster randomized clinical trial was conducted across 4 months (January 1 through April 30, 2023) at a single emergency medical dispatch center serving 1.3 million residents in the Central Denmark Region. Matched pairs of dispatchers were randomized to manage calls using either telephone only communication (control) or video streaming (intervention). Calls were routed to the next available dispatcher who had been idle the longest. Data were analyzed using the intention-to-treat approach.

**Intervention:**

Dispatchers in the intervention group used secure, 1-way smartphone video streaming during emergency calls, while the control group followed standard telephone protocols.

**Main Outcomes and Measures:**

The primary outcome was the percentage of ambulances dispatched at the highest urgency level. Secondary outcomes included 24-hour hospital admissions among initially nonconveyed patients, ICU admission, mortality rates, and dispatch call duration.

**Results:**

Of 18 745 calls (patient median [IQR] age, 57 [31-76] years; 54.4% female), 8124 were received by 10 dispatchers randomized to video streaming and 10 621 by 10 dispatchers randomized to telephone-only communication. Video streaming was established in 3706 of the 8124 calls (45.6% [95% CI, 44.6%-46.7%]). Compared with the control group, the intervention group demonstrated a 5.0% absolute reduction (95% CI, 0.0%-10.1%; *P* = .049) in highest-urgency ambulance dispatches and no significant change (4.5% increase [95% CI, −1.1% to 10.0%; *P* = .11]) in nonconveyance. Hospital admissions among initially nonconveyed patients within 24 hours were reduced by 2.0% (95% CI, −3.7% to −0.2%; *P* = .03). Mean dispatch time was 3.7 minutes (95% CI, 3.4-4.0 minutes) in the video streaming group vs 3.2 minutes (95% CI, 2.9-3.4 minutes) in the telephone-only group, a difference of 0.5 minutes (95% CI, 0.1-0.9 minutes; *P* = .02). Mortality rates at 30 days and ICU admission rates were not statistically different between the 2 groups.

**Conclusions and Relevance:**

In this cluster randomized clinical trial assessing high-stakes emergency calls, video streaming reduced the frequency of highest-urgency ambulance dispatches and lowered hospital admissions among initially nonconveyed patients, but slightly increased dispatch times. These findings suggest that video streaming may improve triage accuracy and reduce hospital and prehospital resource use.

**Trial Registration:**

ClinicalTrials.gov Identifier: NCT05742412

## Key Points

**Question:**

Can video streaming during emergency medical calls enhance triage precision and optimize health resource utilization compared with telephone-only communication?

**Findings:**

This cluster randomized clinical trial of 18 745 emergency calls demonstrated that video streaming reduced the percentage of highest-urgency dispatches by 5.0%, decreased 24-hour hospital admissions among initially nonconveyed patients, and extended call durations by 0.5 minutes (from 3.2 to 3.7 minutes) without any increase in mortality or intensive care unit admission rates.

**Meaning:**

These results suggest that video streaming has the potential to improve triage decision-making and optimize health resource allocation during emergency medical calls, supporting more accurate and efficient prehospital care while maintaining patient safety.

## Teaser

This cluster randomized clinical trial assesses whether video streaming during calls to an emergency medical dispatch center can enhance triage precision and optimize health resource utilization while maintaining patient safety.

## Introduction

Emergency medical services (EMS) dispatchers play a critical role as a first-line contact in emergencies, gathering critical information to ensure that prehospital care is both appropriate and timely.^1,2^ Hospital-based treatments are increasingly shifting to the prehospital phase, which demands an efficient use of the prehospital emergency resources. This need is particularly pressing given the global rise in the requirement for prehospital emergency care, coupled with limited resources in many parts of the world.^3,4^

Video streaming during emergency calls may enhance EMS dispatchers’ situational assessment, enabling more precise triage and resource allocation.^5,6,7^ On-demand video streaming solutions are feasible even in emergency settings and have demonstrated considerable success rates when establishing contact through the caller’s smartphone camera.^8,9,10^ Despite these advancements, there is limited evidence regarding the implementation of video streaming in emergency medical dispatch centers (EMDCs).^11^ Existing studies have primarily focused on the feasibility of video streaming in highly selected populations or as part of simulations.^8,10,11,12,13,14,15,16^ Previous research demonstrates substantial variation in the use of video streaming in clinical practice, with use rates ranging from 1.4% in general emergency calls and 7.7% for patients with mental health conditions to nearly 50% in trauma cases managed by a helicopter EMS.^8,10,15^ Video streaming may improve triage and resource allocation but may also be associated with longer EMS calls.^17^

This cluster randomized clinical trial aimed to investigate whether video streaming, compared with telephone only communication, reduced the number of ambulances dispatched with the highest urgency level. Secondary outcomes included nonconveyance rates, 24-hour hospital admissions among initially nonconveyed patients, call duration, 30-day mortality, and intensive care unit (ICU) admissions, to assess both safety and broader health care system implications.

## Methods

### Trial Design and Oversight

The Dispatch of Emergency Call Using Video Streaming Compared with Traditional Telephone Communication (CAM-VISION) trial was an investigator-initiated, single-center, cluster randomized trial conducted across 4 months, from January 1 through April 30, 2023. The trial aimed to compare 2 modes of communication for EMS dispatchers—video streaming and telephone only communication—and was conducted at the Central Denmark Region’s sole EMDC, which handles emergency calls from 1.3 million inhabitants. Approval was obtained from the Legal Department of the Central Denmark Region and the Danish Data Protection Agency for data storage. The local ethical committee waived the requirement for informed consent, as the study did not fall within the scope of health research requiring ethical approval and involved the implementation of video streaming as a management-approved standard operating procedure. The Statistical Analysis Plan (SAP) was published on ClinicalTrials.gov prior to these approvals (eMethods in Supplement 1). The protocol followed Standard Protocol Items: Recommendations for Interventional Trials (SPIRIT) guidelines, and reporting adhered to the extended Consolidated Standards of Reporting Trials (CONSORT) reporting guideline for cluster randomized trials.^18^ The Trial Protocol and Statistical Analysis Plan (SAP) are available in Supplement 2 (trial registration details are in the eMethods; the CONSORT checklist, in eAppendix 1, and data availability confirmation, in eAppendix 2, all in Supplement 1). The study complied with the Declaration of Helsinki and its amendments.^19^

### Trial Population

In Denmark, all emergency medical calls are handled by EMS dispatchers who are trained as either nurses or paramedics specializing in emergency dispatch. The study included emergency calls managed by dispatchers who were continuously employed throughout the study period (January 1 to April 30, 2023) and had completed a mandatory 6-week training program. Of the 27 dispatchers employed at the EMDC during the study period, 7 were excluded due to incomplete employment, leaving 20 participating dispatchers (10 in each group). Staffing levels consisted of 5 dispatchers during the day, 4 in the evening, and 3 at night. We used a pragmatic setup in which all emergency calls assigned to the participating dispatchers were included.

### Trial Procedures

In Denmark, EMS dispatchers manage emergency calls using the criteria-based Danish Index for Emergency Care (DI), which standardizes response allocation.^20,21,22^ The DI classifies emergency calls into 37 symptom- and incident-based categories, each with subcategories, and outlines 5 urgency levels: level A (life-threatening, immediate response with lights and sirens), level B (urgent, EMS within 30 minutes), level C and level D (nonurgent, interhospital or general practitioner-arranged transfers), and level E (no EMS dispatch). Each call is assigned a DI code to guide resource selection, such as ambulance, paramedic, or physician vehicle, or helicopter. Dispatchers assign urgency levels and allocate resources during the call, adjusting them as new information arises. A technical dispatcher handles operational coordination. The DI protocol supports efficient triage, ensuring rapid response to critical cases and appropriate alternatives for lower-priority situations. The initial urgency level may be revised by EMS providers on scene.^23^

### Intervention

Video streaming was facilitated using Dynamic Infrastructure for Applications and Services, a noncommercial platform developed by the Central Denmark Region for patient consultations. Streaming was initiated immediately after assessing the patient’s consciousness level, before categorizing the situation using the DI and determining the emergency response. However, in predefined urgent scenarios—such as cardiac arrest, traffic collisions, or airway obstructions—streaming began after response allocation. For single-rescuer cardiac arrests, streaming was omitted to prioritize uninterrupted cardiopulmonary resuscitation. Once initiated, a text message with a hyperlink was sent to the caller’s smartphone, allowing the EMS dispatcher to access a real-time, 1-way live video feed via the caller’s smartphone camera without storing any data. This connection was not accessible to on-scene personnel. Dispatchers in the intervention group received a brief introduction to the video platform before trial start and were instructed to use video for all emergency calls during the study. In the control arm, EMS dispatchers adhered to standard telephone communication.

### Randomization

This trial used cluster randomization at the dispatcher level. Matched pairs of dispatchers were created using historical call data from January 1 through March 31, 2022, based on the proportion of highest-urgency dispatches (primary criterion), years of employment, and average call duration. Within each pair, one dispatcher was randomly assigned to the intervention group (video streaming), and the other to the control group (telephone only), corresponding to a block randomization design with blocks of two. Dispatchers hired after this matching period were randomly assigned to either group without pairing.

Incoming emergency calls were automatically routed to the dispatcher who had been idle the longest. As such, patients were allocated to intervention or control group based on which dispatcher answered the call, ensuring unbiased assignment of calls within the randomized cluster design. EMS dispatchers had no access to patient information prior to call initiation. Blinding was not feasible due to the nature of the intervention.

### Outcomes

The primary outcome was the rate of ambulances dispatched at the highest urgency level (A responses), graded on the 5 level dispatch code. Secondary outcomes included the frequency of dispatches across all urgency levels (A, B, C, D, and E), 30-day mortality after study inclusion, the proportion of cases with agreement on urgency levels between EMS dispatchers and EMS providers on the scene, duration from hospital admission to discharge to home or a care facility, and the proportion of patients requiring ICU admission during their hospital stay. Additional secondary outcomes included the proportion of emergency calls with a change in urgency level during the call, changes in allocated resources (eg, ambulance, helicopter, physician-crewed vehicle) compared with initial allocations, 24-hour hospital admission rate among patients initially assigned an E response level and not admitted, total call duration, time from call initiation to dispatch, and EMS on-scene time (from arrival to departure).

### Data Sources

#### EMDC Dispatch System

Data for all emergency calls were retrieved from the EMDC dispatch system, Logis, a computer-aided dispatch system. Information stored in the Logis database included urgency levels, allocated resources, patient and hospital addresses, time stamps for all prehospital activities, and EMS dispatcher identifications (IDs).

#### Research Electronic Data Capture

A secure web-based research database was developed in REDCap to store study-specific data for the video streaming group (intervention).^24^ After each emergency call, EMS dispatchers recorded details about the video call, including whether video streaming was used and, if not, the reason for opting out or the inability to establish a connection. For missing REDCap entries in the video group, call voice logs were reviewed, and video use (attempted, used, or not attempted) was manually registered.

#### Hospital Data

Mortality, hospital length of stay, ICU admissions (department codes), *International Statistical Classification of Diseases, Tenth Revision* (*ICD-10*) discharge diagnoses, and 10-year *ICD-10* diagnosis history for comorbidity assessment were retrieved from the regional Business Intelligence database. Data linkage was achieved using the Danish Social Security Number.

#### Telecommunication Database

The telecommunication database stores all EMDC telephone calls as voice logs. The voice logs also capture the EMS dispatcher ID, call duration, and call date and time.

### Statistical Analysis

The study period, designated by EMS management as a provisional phase before full implementation, yielded a convenience sample. Based on an annual call volume of 60 000 to 80 000,^25,26^ we anticipated 15 000 to 20 000 randomized calls during 4 months—sufficient to evaluate the primary outcome and monitor safety indicators, including mortality, ICU admissions, and hospital admissions occurring within 24 hours after nonconveyance.

The primary analysis followed an intention-to-treat approach, including all patients randomized to video streaming, regardless of whether it was attempted. For binary outcomes, we estimated relative risks (RRs) and risk differences (RDs) using Poisson and linear regression, respectively. For duration outcomes, such as length of stay, we used linear regression for differences in means and generalized linear regression (log-link, gamma distribution) for ratio of means. To account for clustering, all models applied cluster-robust variance estimation, with EMS dispatcher as the cluster unit. The randomized block design was not included in the models, yielding conservative standard errors.

Sensitivity analyses addressed missing data using multiple imputation with chained equations (50 datasets), adjusted for sex, age, and Charlson Comorbidity Index, with restricted cubic splines (3 or 4 knots) for age and Charlson Comorbidity Index. Due to limitations of the telecommunications database—which logs only time, date, and dispatcher ID—some re-calls were included in total call duration estimates. This did not affect the secondary outcome, time from emergency call to dispatch, which was derived from Logis data.

Secondary outcomes were not adjusted for multiple comparisons and should not be interpreted for formal inference. All results are reported with 95% CIs and 2-sided *P* values, with statistical significance defined as *P* < .05. Analyses were conducted using Stata, version 18 (StataCorp LLC).

## Results

During the 4-month study period, the EMDC received 24 943 emergency calls, of which 686 were excluded due to calls being managed by nonparticipating technical dispatchers, which resulted in missing dispatcher IDs in the dataset. An additional 5512 calls were excluded because they were handled by 7 EMS dispatchers not employed for the entire study period. In total, 20 EMS dispatchers were included. Of 18 745 calls (patient data), 8124 were received by 10 dispatchers randomized to video streaming and 10 621 by 10 dispatchers randomized to telephone-only communication (Figure). Baseline characteristics and comorbidities were similar between the 2 groups, as shown in Table 1, and stratified by sex in eTable 1 in Supplement 1. The overall median (IQR) age was 57 (31-76) years, with 54.4% female and 45.6% male. Final diagnoses were also evenly distributed between the intervention and control groups (Table 2).

**Figure.**
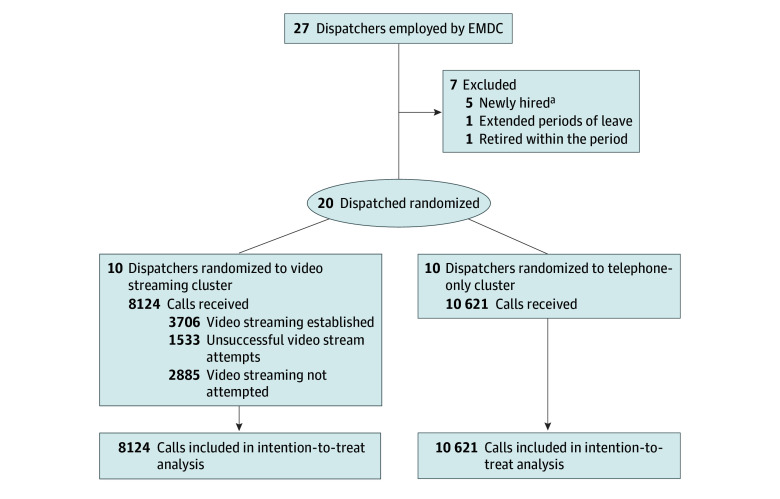
Flowchart of Call Inclusion and Randomization EMDC indicates emergency medical dispatch center. ^a^Included newly hired dispatchers who were not employed for the full study period or who did not complete the mandatory 6-week training.

**Table 1.** Baseline Characteristics and Comorbidities

Characteristic or comorbidity	Calls, No./total No. (%)^a^	
	Video (intervention group)	Telephone only (control group)
Total No.	8124	10 621
Age, median (IQR), y^b^	58 (32-76)	57 (30-75)
Sex^b^		
Female	4054/7528 (53.9)	5365/9814 (54.7)
Male	3474/7528 (46.1%)	4449/9814 (45.3%)
Comorbidities included in the Charlson Comorbidity Index^c^		
Myocardial infarction	335/7528 (4.5)	457/9814 (4.7)
Congestive heart failure	433/7528 (5.8)	561/9814 (5.7)
Peripheral vascular disease	356/7528 (4.7)	438/9814 (4.5)
Cerebrovascular disease	768/7528 (10.2)	975/9814 (9.9)
Hemiplegia	37/7528 (0.5)	50/9814 (0.5)
Dementia	131/7528 (1.7)	153/9814 (1.6)
Chronic pulmonary disease	805/7528 (10.7)	1007/9814 (10.3)
Diabetes (without complications)	334/7528 (4.4)	384/9814 (3.9)
Diabetes (with chronic complications)	287/7528 (3.8)	371/9814 (3.8)
Mild liver disease	187/7528 (2.5)	221/9814 (2.3)
Moderate or severe liver disease	40/7528 (0.5)	47/9814 (0.5)
Connective tissue disease	220/7528 (2.9)	319/9814 (3.3)
Ulcer disease	186/7528 (2.5)	227/9814 (2.3)
Moderate or severe kidney disease	328/7528 (4.4)	434/9814 (4.4)
Any tumor	695/7528 (9.2)	985/9814 (10.0)
Leukemia	24/7528 (0.3)	28/9814 (0.3)
Lymphoma	52/7528 (0.7)	74/9814 (0.8)
Metastatic solid tumor	69/7528 (0.9)	92/9814 (0.9)
AIDS	13/7528 (0.2)	19/9814 (0.2)
Charlson Comorbidity Index^c^		
0	2593/7479 (34.7)	3619/9782 (37.0)
1	825/7479 (11.0)	1023/9782 (10.5)
2	736/7479 (9.8)	893/9782 (9.1)
3	837/7479 (11.2)	1006/9782 (10.3)
4	870/7479 (11.6)	1102/9782 (11.3)
5	603/7479 (8.1)	807/9782 (8.2)
6	413/7479 (5.5)	566/9782 (5.8)
7	262/7479 (3.5)	329/9782 (3.4)
≥8	340/7479 (4.5)	437/9782 (4.5)

^a^ Number of observations divided by total nonmissing data points.

^b^ In 1484 of 18 745 calls (7.9%), the civil registration number was missing, resulting in absent age and sex data (n = 645 for the video group and n = 839 for telephone only).

^c^ Charlson comorbidity index during a 10-year period based on *International Statistical Classification of Diseases, Tenth Revision* codes. In 1403 of 18 745 calls (7.5%), comorbidity data were missing (n = 596 for the video group and n = 807 for the telephone only group).

**Table 2.** Final Diagnosis After Hospital Admission Categorized by *ICD-10* Main Chapters

Characteristic or Diagnosis	Code	Calls, No./total No. (%)^a^	
		Video (intervention group)	Telephone-only (control group)
Admitted to hospital, No.	NA	4354	5977
Age, median (IQR), y^b^	NA	63 (38-77)	62 (36-77)
Sex^b^			
Female	NA	2218/4205 (52.7)	3129/5799 (54.0)
Male	NA	1987/4205 (47.3)	2670/5799 (46.0)
*ICD-10* chapter and code ranges^c^			
Certain Infectious and parasitic diseases	A00-B99	78/4171 (1.9)	119/5728 (2.1)
Neoplasms	C00-D49	8/4171 (0.2)	14/5728 (0.2)
Diseases of the blood and blood-forming organs and certain disorders involving the immune mechanism	D50-D89	15/4171 (0.4)	19/5728 (0.3)
Endocrine, nutritional and metabolic diseases	E00-E89	52/4171 (1.2)	66/5728 (1.2)
Mental, behavioral and neurodevelopmental disorders	F01-F99	169/4171 (4.1)	256/5728 (4.5)
Diseases of the nervous system	G00-G99	131/4171 (3.1)	169/5728 (3.0)
Diseases of the eye and adnexa	H00-H59	3/4171 (0.1)	2/5728 (<0.1)
Diseases of the ear and mastoid process	H60-H95	18/4171 (0.4)	18/5728 (0.3)
Diseases of the circulatory system	I00-I99	395/4171 (9.5)	541/5728 (9.4)
Diseases of the respiratory system	J00-J99	221/4171 (5.3)	347/5728 (6.1)
Diseases of the digestive system	K00-K95	113/4171 (2.7)	175/5728 (3.1)
Diseases of the skin and subcutaneous tissue	L00-L99	8/4171 (0.2)	11/5728 (0.2)
Diseases of the musculoskeletal system and connective tissue	M00-M99	72/4171 (1.7)	114/5728 (2.0)
Diseases of the genitourinary system	N00-N99	95/4171 (2.3)	110/5728 (1.9)
Pregnancy, childbirth and the puerperium	O00-O9A	10/4171 (0.2)	18/5728 (0.3)
Certain conditions originating in the perinatal period	P00-P96	1/4171 (<0.1)	0/5728 (0)
Symptoms, signs, and abnormal clinical and laboratory findings, not elsewhere classified	R00-R99	1383/4171 (33.2)	1963/5728 (34.3)
Injury, poisoning, and certain other consequences of external causes	S00-T88	1000/4171 (24.0)	1293/5728 (22.6)
Factors influencing health status and contact with health services	Z00-Z99	399/4171 (9.6)	493/5728 (8.6)

Abbreviations: ICD-10, *International Statistical Classification of Diseases, Tenth Revision*; NA, not applicable.

^a^ Number of observations divided by total nonmissing data points.

^b^ In 327 of 10 331 admissions (3.2%), the civil registration number was missing, resulting in missing age and sex data (n = 149 for the video group and n = 178 for telephone only).

^c^ In 432 of 10 331 admissions (4.2%), the primary *ICD-10* diagnoses were missing in the hospital registry (n=183 for the video group, and n=249 for telephone only group).

Of the 8124 calls in the intervention group, video streaming was successfully established in 3706 cases (45.6% [95% CI, 44.6%-46.7%]), attempted but unsuccessful in 1533 cases (18.9% [95% CI, 18.0%-19.7%]), and not attempted in 2885 cases (35.5% [95% CI, 34.5%-36.6%]). Dispatcher-reported reasons were available for 1326 of the 1533 unsuccessful attempts and for 1126 of the 2885 cases in which video streaming was not attempted (Table 3). The most common reasons for unsuccessful video streaming were phone limitations, technical issues with SMS (short message service) or link setup, and uncooperative callers, totaling 93.1% of cases. For calls in which video streaming was not attempted, the top reasons included communication barriers, the caller not being physically present with the patient, high call volume at the EMDC, and estimated time of arrival re-calls, accounting for 81.8% (Table 3).

**Table 3.** Dispatcher-Reported Reasons for Video Streaming Not Being Initiated or Not Successfully Established in the Video Streaming Group

Description	No. of calls	Percentage of calls	Cumulative percentage
Dispatcher-reported reasons for video streaming not being attempted			
Communication challenges, including language barriers	481	42.7	42.7
Caller not physically present with the patient	227	20.2	62.9
High call volume at Emergency Medical Dispatch Center	110	9.8	72.7
Re-call regarding estimated time of arrival	103	9.2	81.8
Caller alone performing CPR	52	4.6	86.4
Not used due to short ambulance response time	33	2.9	89.4
Not assessed as an emergency	32	2.8	92.2
Other, unspecified	26	2.3	94.5
Dispatcher forgot to initiate video	20	1.8	96.3
Technical difficulties	13	1.2	97.4
Urological or gynecological condition	11	1.0	98.4
No caller on the line	9	0.8	99.2
Frequent caller or known repeated contact	6	0.5	99.8
Unmistakable signs of death	3	0.3	100
Total	1126	100	NA
Missing dispatcher-reported reasons in REDCap	1759	NA	NA
Reported reasons for unsuccessful video streaming attempts			
Phone not suitable, including landline, prepaid, or international SIM	429	32.4	32.4
Technical failure during SMS or link setup	420	31.7	64.0
Caller unable to cooperate during video setup	386	29.1	93.1
Caller declined to initiate video connection	51	3.9	97.0
Unstable or weak network connection	22	1.7	98.6
Visual conditions too poor for video (eg, darkness)	6	0.5	99.1
Other or unsuitable for video communication	12	0.9	100
Total	1326	100	NA
Missing dispatcher-reported reasons in REDCap	207	NA	NA

Abbreviations: CPR, cardiopulmonary resuscitation; NA, not applicable; SIM, subscriber identity module; SMS, short message service.

eTable 2 in Supplement 1 provides detailed data on video streaming establishment, stratified by primary symptom or incident categories as defined in the DI. Streaming was most frequently established in cases involving children (76.2%), seizures (65.8%), airway obstruction (61.1%), unconscious adults (56.0%), trauma-related incidents excluding traffic collisions (54.4%), and traffic collisions (54.2%). Moderate rates were observed in reduced consciousness or paralysis (51.8%), chest pain (40.1%), and respiratory distress (39.7%). Psychiatric cases had the lowest rate among common presentations (18.2%).

Of 18 745 patients, 8415 were not admitted to the hospital (nonconveyance). Nonconveyance decisions were made during emergency calls in 5248 cases (E level response) and at the scene in 3167 cases. Nonconveyance at the scene accounted for 20.6% (95% CI, 19.7%-21.6%) of all A level responses and 26.2% (95% CI, 25.1%-27.3%) of all B level responses (eFigure in Supplement 1).

### Primary Outcome

The percentage of calls classified at the highest urgency level (A level responses) was 38.4% (95% CI, 35.5%-41.2%) in the control group (telephone only) and 33.3% (95% CI, 29.6%-37.0%) in the intervention group (video streaming) (Table 4). This finding represented a statistically significant absolute reduction of 5.0 percentage points (95% CI, 0.0-10.1 percentage points; *P* = .049) and a relative reduction of 13.2% (95% CI, 0.6%-24.1%; *P* = .04) in A level responses.

**Table 4.** Primary and Secondary Outcomes

Outcome	Percentage of calls (95% CI)		Risk difference (95% CI)^a^	Relative risk (95% CI)^a^	Missing, No.
	Video (intervention group)	Telephone-only (control group)			
Primary outcome					
Highest level of urgency rate (response A)^b^^,^^c^	33.3 (29.6 to 37.0)	38.4 (35.5 to 41.2)	−5.0 (−10.1 to −0.0)	0.87 (0.76 to 0.99)	0
No.	2706	4074	NA	NA	NA
Secondary binary outcomes					
Second highest level of urgency (response B)^b^^,^^c^	36.1 (33.0 to 39.1)	35.5 (33.1 to 37.8)	0.6 (−3.5 to 4.7)	1.02 (0.91 to 1.13)	0
No.	2930	3766	NA	NA	NA
Lowest level of urgency (nonconveyance dispatch, response E)^b^^,^^c^	30.5 (26.5 to 34.5)	26.1 (22.8 to 29.3)	4.5 (−1.1 to 10.0)	1.17 (0.98 to 1.41)	0
No.	2480	2768	NA	NA	NA
30-d Mortality^b^	2.7 (2.4 to 3.0)	2.7 (2.4 to 2.9)	0.1 (−0.3 to 0.4)	1.02 (0.89 to 1.17)	0
No.	221	283	NA	NA	NA
No. of emergency calls with changed urgency level during dispatch^b^	6.8 (5.4 to 8.1)	6.9 (5.4 to 8.4)	−0.2 (−2.3 to 2.0)	0.98 (0.73 to 1.31)	3
No.	549	734	NA	NA	NA
No. of emergency calls with urgency level subsequently increased during dispatch^b^	1.8 (1.4 to 2.3)	2.5 (1.7 to 3.4)	−0.7 (−1.7 to 0.3)	0.72 (0.48 to 1.10)	3
No.	149	269	NA	NA	NA
No. of emergency calls with urgency level subsequently lowered during dispatch^b^	4.9 (3.7 to 6.1)	4.4 (3.5 to 5.2)	0.5 (−1.0 to 2.1)	1.13 (0.82 to 1.54)	3
No.	400	465	NA	NA	NA
No. of emergency calls with changed resource allocation during dispatch^b^	9 (7.6 to 10.4)	8.3 (6.4 to 10.2)	0.7 (−0.2 to 3.2)	1.09 (0.83 to 1.43)	356
No.	720	865	NA	NA	NA
Percentage of identical urgency levels for ambulance transports to and from the scene^d^	47.4 (44.3 to 50.4)	43.5 (41.7 to 45.4)	4.0 (0.1 to 7.6)	1.09 (1.01 to 1.18)	509
No.	2561	3302	NA	NA	NA
No. of participants requiring ICU admission at the hospital^e^	0.6 (0.4 to 0.9)	0.6 (0.3 to 0.8)	0.1 (−0.3 to 0.4)	1.17 (0.68 to 2.00)	0
No.	28	33	NA	NA	NA
24-h Admissions among nonconveyed patients ≤24 h after response level E (nonconveyance)^f^	8.5 (7.3 to 9.7)	10.5 (9.3 to 11.6)	−2 (−3.7 to −0.2)	0.81 (0.68 to 0.97)	839
No.	181	239	NA	NA	NA
Secondary duration outcomes					
Time from emergency call to dispatch, min^b^	3.7 (3.4 to 4.0)^g^	3.2 (2.9 to 3.4)^g^	0.5 (0.1 to 0.9)^a^^,^^h^	1.16 (1.04 to 1.30)^a^^,^^i^	3
On-scene time, min^d^	21.4 (21.0 to 21.9)^g^	20.9 (20.5 to 21.3)^g^	0.6 (−0.0 to 1.2)^a^^,^^h^	1.03 (1.00 to 1.06)^a^^,^^i^	3196
Length of stay at hospital, d^e^	0.54 (0.48 to 0.61)^g^	0.52 (0.49 to 0.55)^g^	0 (−0.0 to 0.1)^a^^,^^h^	1.05 (0.93 to 1.20)^a^^,^^i^	425
Duration of emergency medical calls, min^j^	4.1 (3.6 to 4.5)	3.8 (3.2 to 4.4)	0.3 (−0.4 to 1.0)^a^^,^^h^	1.07 (0.90 to 1.28)^a^^,^^i^	0

Abbreviations: EMS, emergency medical services; ICU, intensive care unit; NA, not applicable.

^a^ Telephone only (control group) as reference.

^b^ Population A, the total study population.

^c^ For details on subpopulations indicated with response level A, B, C, D, and E see the extended flowchart in eFigure in Supplement 1.

^d^ Population B, cases assessed on-scene by EMS providers.

^e^ Population C, cases admitted to hospital.

^f^ Population D, cases without EMS resource allocation after the emergency call (E level responses).

^g^ Mean (95% CI).

^h^ Difference in means (95% CI).

^i^ Ratio of means (95% CI).

^j^ Population E, all EMS calls answered by EMS dispatchers during the study period (n = 20 722); higher call volume compared with the study population (n = 1977) is attributed to re-calls that cannot be distinguished from primary calls in the telephone call data.

### Secondary Outcomes

The difference in dispatched A level responses did not affect the proportion of B level responses (second-highest urgency). However, in the video streaming group, there was no significant change (4.5 percentage points [95% CI, −1.1 to 10.0 percentage points; *P* = .11]) in E level responses in which no EMS response was dispatched (nonconveyance) (Table 4). This was accompanied by a significant reduction of 2.0 percentage points (95% CI, −3.7 to −0.2 percentage points; *P* = .03) in 24-hour admissions among nonconveyed patients and an increase of 4.0 percentage points (95% CI, 0.1-7.6 percentage points; *P* = .047) in the percentage of identical urgency levels for ambulances dispatched to and from the scene.

The 30-day mortality rate (2.7%) and ICU admission rates (0.6%) were identical in both groups, with a risk difference of 0.1 percentage points (95% CI, −0.3 to 0.4 percentage points) for both factors (*P* = .76 and *P* = .58, respectively). The mean hospital length of stay showed minimal variation, with a difference of 0.03 days (95% CI, −0.05 to 1.00 days; *P* = .44).

The mean call duration was 4.1 (95% CI, 3.6-4.5) minutes in the video group and 3.8 (95% CI, 3.2-4.4) minutes in the telephone-only group. Time from call to dispatch was 3.7 (95% CI, 3.4-4.0) minutes in the video group vs 3.2 (95% CI, 2.9-3.4) minutes in the telephone-only group, a difference of 0.5 (95% CI, 0.1-0.9) minutes (*P* = .02). On-scene times were similar: 21.4 (95% CI, 21.0-21.9) minutes in the video group vs 20.9 (95% CI, 20.5-21.3) minutes in the telephone only group.

Sensitivity analyses supported the primary findings, with no changes in interpretation (eTable 3 in Supplement 1). Frequencies of A level response dispatches by primary symptoms used for triaging are provided in eTable 4 in Supplement 1.

## Discussion

This cluster randomized study demonstrated that incorporating video streaming in EMS dispatch, compared with telephone-only communication, was associated with a significant 5.0 percentage point reduction in highest urgency transports, a 2.0 percentage point reduction in 24-hour admissions among nonconveyed patients, and a nonsignificant 4.5 percentage point increase in nondispatches. Mortality and ICU admission rates were similar between groups. Video streaming also improved triage accuracy, with a 4.0 percentage point increase in identical urgency levels for dispatched and return transports. Calls were a mean of 0.5 minutes longer when video was used.

To our knowledge, this is the first large cluster randomized clinical trial comparing video streaming and telephone only triage by EMS dispatchers. While the video streaming success rate may appear modest, it reflects feasibility under routine operational conditions. All emergency calls were included around the clock, without preselection, capturing a broad range of situations in which video was impractical or inappropriate—such as when the caller was not with the patient, communication was limited, the call was a re-contact for estimated time of arrival, or the caller was alone performing cardiopulmonary resuscitation.

To our knowledge, the only other study including unselected emergency calls was by Linderoth et al,^8^ in which dispatchers could add live video but had to determine the emergency response before initiating it. Video streaming was successfully established in 1.1% of all calls (838 of 73 442) and led to a change in dispatcher assessment in 51.1% of cases and in emergency response in 27.5%, yielding higher odds of response change compared with a historical control. These findings highlight both the potential impact of video and the challenges of integrating it into standard operations.

Other studies on video streaming and dispatch have examined its use in selected calls. In a retrospective Swiss study,^27^ 20.1% of suspected COVID-19 cases were referred to second-line physicians, with successful video streaming in 17.5% and reported aid to decision-making in 75.7% of these cases. In the present study, by contrast, video was successfully established in 39.7% of first-line calls involving respiratory distress—a high first-line success rate (eTable 2 in Supplement 1).

In the SEE-IT trial involving trauma calls referred to a helicopter EMS tasking desk,^10^ video was successfully established in 48.2% of intervention arm incidents. It enhanced situational awareness and informed dispatch decisions.

Finally, a study involving secondary triage of patients with mental health problems reported a 7.7% use of video streaming, associated with reduced EMS dispatches.^15^ In the present study, video streaming had the lowest usage rate among psychiatric cases, with successful establishment in 18.2% of cases.

While previous studies focused on selected cases managed by second-line or specialist personnel, they support the feasibility and clinical value of video streaming, with a general tendency to reduce highest urgency in time-critical EMS settings. The impact of video streaming likely varies by clinical context. Prior studies have suggested benefits in calls involving children, cardiac arrest, neurological symptoms, and trauma.^8,13,14^ In line with these findings, the present study observed high video use in similar cases.

### Limitations

This study has limitations. First, the acute setting made it difficult to obtain personal identification for all callers, reducing data completeness. Second, the slightly longer EMS call duration with video streaming may have led to fewer intervention-group calls during busy periods. If dispatcher availability was influenced by workload, this could have introduced confounding. Some added call time may also reflect trial procedures, such as after call data entry in REDCap. Third, the lack of ethnicity or race data and the single-center design limit generalizability. Additionally, video streaming was only successfully established in 3706 of 5239 attempted cases, reflecting routine operational challenges with a simple, noncommercial 1-way video platform. Future technological advancements may increase success rates, but cost-effectiveness and wider implementation in routine practice remain uncertain due to training needs, costs, and longer call times.

## Conclusions

This cluster randomized clinical trial found that incorporating video streaming in addition to telephone communication significantly reduced ambulances dispatched at the highest urgency level and lowered 24-hour admissions among nonconveyed patients, both indicating improved resource allocation. A modest but significant increase in time from call to dispatch was observed. These findings underscore the potential of video streaming to enhance triage precision and support more efficient emergency care delivery. Further research into its cost-effectiveness and scalability is warranted.

## Notes

Supplement 1. **eFigure.** Extended flowchart displaying subgroups for primary and secondary outcomes analyses **eTable 1.** Baseline characteristics and comorbidities stratified by sex and randomized group **eTable 2.** Proportion of cases with video streaming established across Danish Index dispatch chapters (primary symptom or incident category identified during the emergency call) **eTable 3.** Adjusted analyses for results with missing outcome (multiple imputation) **eTable 4.** Proportion of highest urgency (level A) in emergency medical calls: video intervention versus telephone-only control, based on single chapters from the Danish Index dispatch reference **eMethods.** Trial registration **eAppendix 1.** CONSORT 2010 Checklist for Cluster Randomized Trials **eAppendix 2.** CAM-VISION trial data availability confirmation

Supplement 2.
Trial Protocol

Supplement 3.
Data Sharing Statement
