# Supplementary material for: Video Streaming or Telephone Communication During Emergency Medical Services Dispatch Calls: A Cluster Randomized Clinical Trial
Source: JAMA Netw Open. 2025 Jul 1;8(7):e2519020. doi: 10.1001/jamanetworkopen.2025.19020 (PMC12215568; doi:10.1001/jamanetworkopen.2025.19020)
Supplement: Supplement 1. — eFigure. Extended flowchart displaying subgroups for primary and secondary outcomes analyses eTable 1. Baseline characteristics and comorbidities stratified by sex and randomized group eTable 2. Proportion of cases with video streaming established across Danish Index dispatch chapters (primary symptom or incident category identified during the emergency call) eTable 3. Adjusted analyses for results with missing outcome (multiple imputation) eTable 4. Proportion of highest urgency (level A) in emergency medical calls: video intervention versus telephone-only control, based on single chapters from the Danish Index dispatch reference eMethods. Trial registration eAppendix 1. CONSORT 2010 Checklist for Cluster Randomized Trials eAppendix 2. CAM-VISION trial data availability confirmation [file jamanetwopen-e2519020-s001.pdf]

Supplementary Online Content

Gude MF, Valentin JB, Meisner-Jensen M, et al. Video streaming or telephone communication during emergency medical services dispatch calls: a cluster randomized clinical trial. *JAMA Netw Open*. 2025;8(7):e2519020. doi:10.1001/jamanetworkopen.2025.19020

eFigure. Extended flowchart displaying subgroups for primary and secondary outcomes analyses
eTable 1. Baseline characteristics and comorbidities stratified by sex and randomized group
eTable 2. Proportion of cases with video streaming established across Danish Index dispatch chapters (primary symptom or incident categories identified during the emergency call)
eTable 3. Adjusted analyses for results with missing outcome (multiple imputation)
eTable 4. Proportion of highest urgency (level A) in emergency medical calls: video intervention versus telephone-only control, based on single chapters from the Danish Index dispatch reference
eMethods. Trial registration
eAppendix 1. CONSORT 2010 Checklist for Cluster Randomized Trials
eAppendix 2. CAM-VISION trial data availability confirmation

This supplementary material has been provided by the authors to give readers additional information about their work.

© 2025 Gude MF et al. *JAMA Network Open*.

eFigure. Extended flowchart displaying subgroups for primary and secondary outcomes analyses

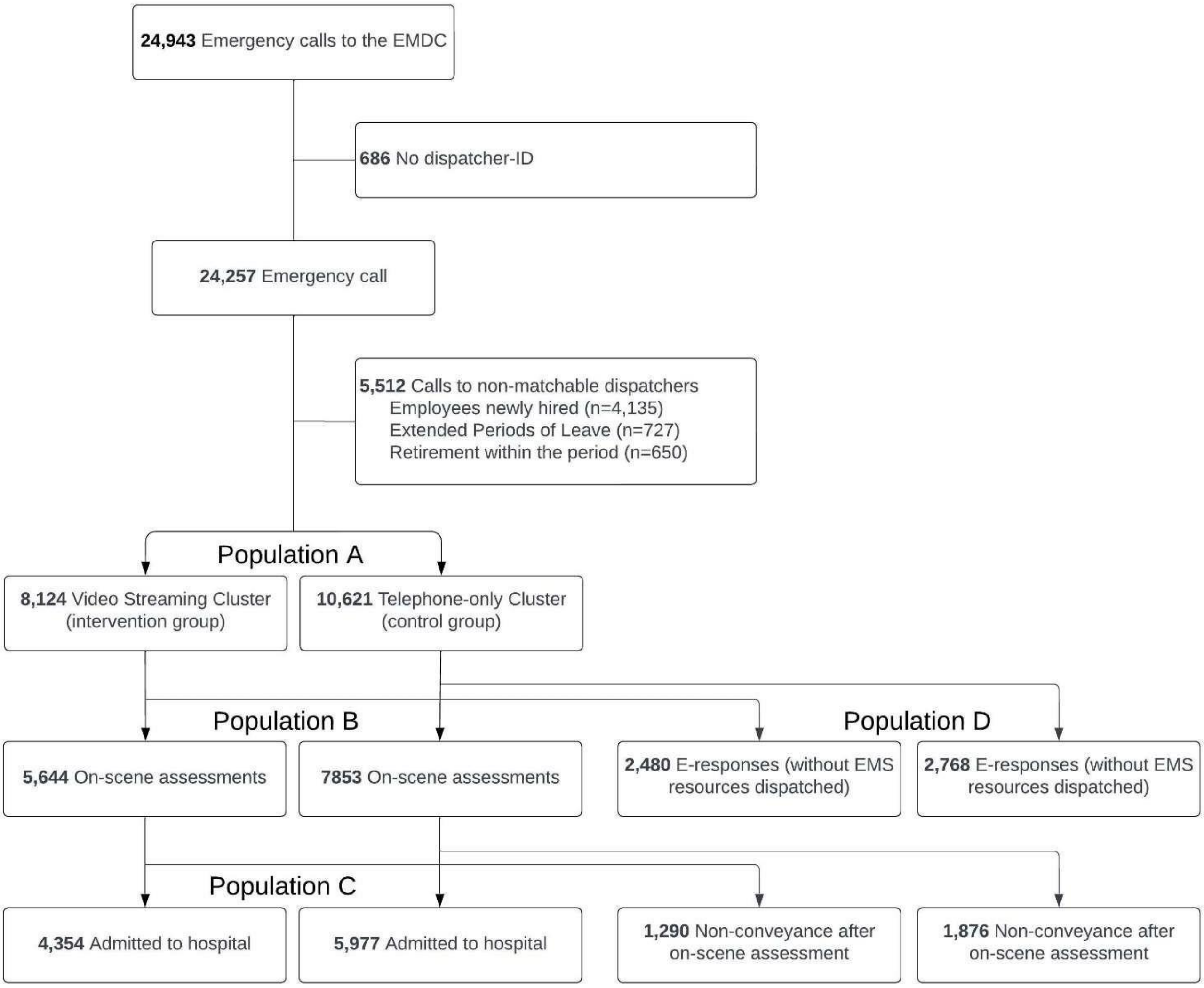

Population A: Total study population.

Population B: Cases assessed on scene by EMS providers (excluding Level E responses without EMS dispatch).

Population C: Cases admitted to hospital (excluding non-conveyed patients after EMS on-scene assessment).

Population D: Cases without EMS resource allocation after the emergency call (E-responses).

Abbreviations: EMS: Emergency medical services.

© 2025 Gude MF et al. *JAMA Network Open*.

eTable 1. Baseline characteristics and comorbidities stratified by sex and randomized group

	Male		Female	
**Factor**	**Video**	**Telephone**	**Video**	**Telephone**
N	3447	4434	4032	5348
Age, median (IQR)[a]	58 (31, 77)	56 (28, 76)	58 (34, 75)	57 (33, 74)
**Comorbidities included in the Charlson Comorbidity Index, n/N (%)[b]**				
Myocardial infarction	104 (3.0)	142 (3.2)	228 (5.7)	313 (5.9)
Congestive heart failure	139 (4.0)	167 (3.8)	291 (7.2)	390 (7.3)
Peripheral vascular disease	111 (3.2)	148 (3.3)	243 (6.0)	288 (5.4)
Cerebrovascular disease	313 (9.1)	397 (9.0)	453 (11.2)	576 (10.8)
Hemiplegia	14 (0.4)	21 (0.5)	23 (0.6)	29 (0.5)
Dementia	56 (1.6)	68 (1.5)	74 (1.8)	85 (1.6)
Chronic pulmonary disease	388 (11.3)	461 (10.4)	412 (10.2)	542 (10.1)
Diabetes mellitus (without complications)	138 (4.0)	193 (4.4)	80 (2.0)	122 (2.3)
Diabetes mellitus (with chronic complications)	81 (2.3)	83 (1.9)	105 (2.6)	144 (2.7)
Mild liver disease	85 (2.5)	95 (2.1)	101 (2.5)	126 (2.4)
Moderate/severe liver disease	13 (0.4)	20 (0.5)	27 (0.7)	27 (0.5)
Connective tissue disease	125 (3.6)	152 (3.4)	209 (5.2)	231 (4.3)
Ulcer disease	88 (2.6)	115 (2.6)	199 (4.9)	255 (4.8)
Moderate/severe renal disease	111 (3.2)	150 (3.4)	216 (5.4)	282 (5.3)
Any tumor	298 (8.6)	401 (9.0)	393 (9.7)	578 (10.8)
Leukemia	3 (0.1)	8 (0.2)	21 (0.5)	20 (0.4)
Lymphoma	23 (0.7)	25 (0.6)	29 (0.7)	49 (0.9)
Metastatic solid tumor	32 (0.9)	44 (1.0)	36 (0.9)	48 (0.9)
AIDS	2 (0.1)	5 (0.1)	11 (0.3)	14 (0.3)
**Charlson Comorbidities Index, n/N (%)[b]**				
Score 0	1220 (35.4)	1710 (38.6)	1373 (34.1)	1909 (35.7)
Score 1	378 (11.0)	465 (10.5)	446 (11.1)	554 (10.4)
Score 2	313 (9.1)	343 (7.7)	420 (10.4)	547 (10.2)
Score 3	402 (11.7)	446 (10.1)	427 (10.6)	563 (10.5)
Score 4	417 (12.1)	539 (12.2)	455 (11.3)	560 (10.5)
Score 5	309 (9.0)	381 (8.6)	298 (7.4)	423 (7.9)
Score 6	187 (5.4)	253 (5.7)	224 (5.6)	317 (5.9)
Score 7	97 (2.8)	134 (3.0)	165 (4.1)	195 (3.6)
Score ≥8	124 (3.6)	163 (3.7)	224 (5.6)	280 (5.2)

Data are presented for patients in the video streaming (intervention) and telephone-only (control) groups, stratified by sex.
Charlson comorbidities index over a 10-year period based on ICD-10 codes.
n/N: Number of observations (n) over the total non-missing data points (N)

[a]In 1,484 of 18,745 calls (7.9%), the civil registration number (CRN) was missing, resulting in absent age and sex data (n=645 for the video group and n=839 for telephone only). [b]In 1,403 of 18,745 calls (7.5%) comorbidity data was missing

© 2025 Gude MF et al. *JAMA Network Open*.

(n=596 for the video group and n=807 for the telephone only group).

No significant differences were observed in baseline characteristics or comorbidities between the groups.

© 2025 Gude MF et al. *JAMA Network Open*.

eTable 2. Proportion of cases with video streaming established across Danish Index dispatch chapters (primary symptom or incident categories identified during the emergency call)

Dispatch reference chapter	Number of observations (n)	Proportion of cases with video streaming attempted	Proportion of cases with video streaming established
01 Unconscious adult (post-puberty)	191	76.4%	56.0%
03 Airway obstruction by a foreign object	18	77.8%	61.1%
06 Unknown problem	760	48.0%	31.1%
07 Allergic reaction	54	72.2%	51.9%
08 Bleeding - non-traumatic	160	60.6%	38.1%
09 Burn - electrical injury	45	40.0%	31.1%
10 Chest pain - heart disease	817	60.1%	40.1%
11 Diabetes mellitus conditions	92	73.9%	50.0%
15 Fever	126	65.9%	44.4%
18 Gynecology - pregnancy	34	50.0%	32.4%
19 Headache	95	68.4%	48.4%
21 Hypothermia - hyperthermia	14	42.9%	14.3%
23 Seizures	409	81.2%	65.8%
24 Abdominal pain - back pain	609	58.0%	41.1%
25 Deceased (not reversible cardiac arrest)	117	62.4%	40.2%
26 Reduced consciousness - paralysis - dizziness	1107	73.3%	51.8%
27 Psychiatry - suicidal	341	29.0%	18.2%
28 Respiratory distress	559	63.1%	39.7%
29 Alcohol - poisoning - overdose	403	58.1%	42.7%
30 Child with medical concern	126	86.5%	76.2%

© 2025 Gude MF et al. *JAMA Network Open*.

Dispatch reference chapter	Number of observations (n)	Proportion of cases with video streaming attempted	Proportion of cases with video streaming established
31 Extremity pain - wound - fracture – minor injuries	496	71.2%	52.0%
32 Traffic accident	295	69.8%	54.2%
33 Accidents (excluding traffic accidents)	1010	73.9%	54.4%
34 Urinary tract conditions	40	42.5%	32.5%
35 Violence - abuse	40	75.0%	45.0%
36 Ear – nose – throat conditions	67	65.7%	44.8%
37 Eye conditions	18	55.6%	22.2%
Low-frequency chapters*	32	59.4%	40.6%
Missing data in the Danish Index dispatch reference	49	77.6%	51.0%
Total	8124	64.5%	45.6%

*Low-frequency dispatch chapters include those with fewer than 10 observations: Unconscious child (pre-puberty), Drowning, Animal bite/insect sting, Childbirth, Skin and rash, and Chemicals/gases.

© 2025 Gude MF et al. *JAMA Network Open*.

eTable 3. Adjusted analyses for results with missing outcome (multiple imputation)

	Video group, % (95% CI)	Telephone-only group, % (95% CI)	RD (95% CI)*	RR (95% CI)*	Missing, n
**Secondary binary outcomes**					
Number of emergency calls with changed urgency level during dispatch[A]	6.8 (5.4; 8.1)	6.9 (5.4; 8.4)	-0.1 (-2.3; 2.0)	0.981 (0.733; 1.314)	3
Number of emergency calls with urgency level subsequently increased during dispatch[A]	1.8 (1.4; 2.3)	2.5 (1.7; 3.4)	-0.7 (-1.7; 0.3)	0.723 (0.476; 1.097)	3
Number of emergency calls with urgency level subsequently lowered during dispatch[A]	4.9 (3.7; 6.2)	4.4 (3.5; 5.2)	0.6 (-1.0; 2.2)	1.131 (0.826; 1.549)	3
Number of emergency calls with changed resource allocation during dispatch[A]	9.1 (7.7; 10.4)	8.3 (6.4; 10.1)	0.8 (-1.7; 3.2)	1.092 (0.833; 1.431)	356
Proportion of identical urgency levels for ambulance transports to and from the scene[B]	47.3 (44.3; 50.2)	43.6 (41.8; 45.4)	3.7 (-0.1; 7.4)	1.084 (1.006; 1.168)	509
Readmission rate ≤24 hours after response level E (non-conveyance)[D]	8.6 (7.4; 9.7)	10.4 (9.2; 11.6)	-1.8 (-3.7; 0.0)	0.824 (0.688; 0.986)	839
	**Video group, mean (95% CI)**	**Telephone-only group, mean (95% CI)**	**DiM (95% CI)***	**RoM (95% CI)***	**Missing, n**
**Secondary duration outcomes**					
Time from emergency call to dispatch, minutes[A]	3.7 (3.4; 4.0)	3.2 (2.9; 3.4)	0.5 (0.1; 0.9)	1.167 (1.042; 1.306)	3
On-scene time, minutes[B]	21.3 (20.8; 21.8)	20.8 (20.4; 21.1)	0.5 (-0.1; 1.1)	1.024 (0.998; 1.051)	3,196
Length of stay at hospital, days[C]	0.54 (0.47; 0.61)	0.52 (0.49; 0.55)	0.0 (-0.1; 0.1)	1.027 (0.907; 1.164)	425

[A] = Population A - total study population.
[B] = Population B - cases assessed on-scene by EMS providers.
[C] = Population C - cases admitted to hospital.
[D] = Population D - cases without EMS resource allocation after the emergency call (E-responses).
[E] = Population E - all emergency medical service calls answered by EMS dispatchers during the study period including recalls (n = 20,722).

© 2025 Gude MF et al. *JAMA Network Open*.

eTable 4. Proportion of highest urgency (level A) in emergency medical calls: video intervention versus telephone-only control, based on single chapters from the Danish Index dispatch reference

**Description**	**Video group (intervention)**			**Telephone-only group (control)**		
	n/N	Proportion of level A	CI	n/N	Proportion of level A	CI
**Unconscious adult (post-puberty)**	190/191	0.995	0.964–0.999	214/214	1.00	n/a
**Airway obstruction by a foreign object**	7/18	0.389	0.198–0.621	23/36	0.639	0.473–0.777
**Unknown problem**	231/760	0.304	0.272–0.338	272/765	0.356	0.322–0.390
**Allergic reaction**	29/54	0.537	0.405–0.664	48/88	0.545	0.441–0.646
**Bleeding - non-traumatic**	49/160	0.306	0.240–0.382	95/215	0.442	0.377–0.509
**Burn - electrical injury**	22/45	0.489	0.348–0.632	25/51	0.490	0.357–0.625
**Chest pain - heart disease**	596/817	0.729	0.698–0.759	1045/1315	0.795	0.772–0.816
**Diabetes**	39/92	0.424	0.327–0.527	52/112	0.464	0.374–0.557
**Fever**	4/126	0.032	0.012–0.082	23/134	0.172	0.117–0.245
**Gynecology - pregnancy**	11/34	0.324	0.189 – 0.495	14/49	0.286	0.177–0.426
**Headache**	28/95	0.295	0.212–0.394	47/121	0.388	0.306–0.478
**Hypothermia - hyperthermia**	7/14	0.500	0.260–0.740	3/11	0.273	0.090–0.586
**Seizures**	240/409	0.587	0.538–0.634	318/538	0.591	0.549–0.632
**Abdominal pain - back pain**	43/609	0.071	0.053–0.094	54/823	0.066	0.051–0.085
**Found deceased (not witnessed cardiac arrest)**	46/117	0.393	0.309–0.484	54/103	0.524	0.428–0.619

© 2025 Gude MF et al. *JAMA Network Open*.

Description	Video group (intervention)			Telephone-only group (control)		
**Reduced consciousness - paralysis - dizziness**	501/1107	0.453	0.424–0.483	832/1544	0.539	0.514–0.564
**Psychiatry - suicidal**	25/341	0.073	0.050–0.106	43/470	0.091	0.069–0.121
**Respiratory distress**	170/559	0.304	0.267–0.344	275/814	0.338	0.306–0.371
**Alcohol - poisoning - overdose**	43/403	0.107	0.080–0.141	96/621	0.155	0.128–0.185
**Child with medical concern**	27/126	0.214	0.151–0.294	78/185	0.422	0.353–0.494
**Extremity pain - wound - fracture - minor injuries**	20/496	0.040	0.026–0.062	27/635	0.043	0.029–0.061
**Traffic accident**	196/295	0.664	0.609–0.716	199/267	0.745	0.690–0.794
**Accidents (excluding traffic)**	123/1010	0.122	0.103–0.143	150/1145	0.131	0.113–0.152
**Urinary tract conditions**	0/40	0	n/a	0/56	0	n/a
**Violence - abuse**	12/40	0.300	0.179–0.457	17/83	0.205	0.131–0.305
**Ear - nose – throat conditions**	0/67	0	n/a	5/100	0.050	0.021–0.115
**Eye conditions**	2/18	0.111	0.028–0.352	0/19	0	n/a
**Low-frequency chapters**	18/32	0.563	0.390–0.721	25/47	0.532	0.391–0.668
**Missing data in the Danish Index dispatch reference**	26/49	0.531	0.411–0.688	41/60	0.678	0.549–0.784
**Total**	2706/8124	0.333	0.296–0.371	4078/10621	0.384	0.355–0.412

Low-frequency chapters = chapters with less than 10 observations (Unconscious child (pre-puberty); Drowning; Animal bite - insect sting; Poisoning in children; Childbirth; Skin and rash; Chemicals – gases)

© 2025 Gude MF et al. *JAMA Network Open*.

eMethods. Trial registration

The trial was registered on ClinicalTrials.gov 12 days after the enrollment of the first patient (January 1, 2023, at 12:01:32 AM). While this registration occurred later than recommended, the study adhered to Danish legislation and ethical guidelines, which waived the requirement for informed consent, as confirmed by the Ethical Committee (case ID: 1-10-72-6-23). Under these regulations, prospective data collection was not permitted, and the research team exclusively monitored video usage rates as part of the intervention implementation.

The finalized Statistical Analysis Plan (SAP) was signed on August 27, 2023, one day before data access permission was granted (August 28, 2023). Until that date, no data—including prehospital or in-hospital outcome data—was accessible to any member of the research team, as confirmed by the Legal Office of the Central Denmark Region (CAM-VISION Trial Data Availability Confirmation – Letter included at the end of this document).

This ensured that the study design, conduct, and analysis plan remained unbiased and uninfluenced by interim or outcome data, maintaining the integrity of the research process.

© 2025 Gude MF et al. *JAMA Network Open*.

eAppendix 1. CONSORT 2010 Checklist for Cluster Randomized Trials

Section/topic and item No	Standard checklist item	Extension for cluster designs	Page No*
**Title and abstract**			
1a	Identification as a randomised trial in the title	Identification as a cluster randomised trial in the title	1
1b	Structured summary of trial design, methods, results, and conclusions (for specific guidance see CONSORT for abstracts)[11 12]	See table 2	3-4
**Introduction**			
Background and objectives:			
2a	Scientific background and explanation of rationale	Rationale for using a cluster design	5-6
2b	Specific objectives or hypotheses	Whether objectives pertain to the cluster level, the individual participant level, or both	5
**Methods**			
Trial design:			
3a	Description of trial design (such as parallel, factorial) including allocation ratio	Definition of cluster and description of how the design features apply to the clusters	6-8
3b	Important changes to methods after trial commencement (such as eligibility criteria), with reasons		na
Participants:			
4a	Eligibility criteria for participants	Eligibility criteria for clusters	6-7
4b	Settings and locations where the data were collected		6-10
Interventions:			
5	The interventions for each group with sufficient details to allow replication, including how and when they were actually administered	Whether interventions pertain to the cluster level, the individual participant level, or both	6-9
Outcomes:			
6a	Completely defined prespecified primary and secondary outcome measures, including how and when they were assessed	Whether outcome measures pertain to the cluster level, the individual participant level, or both	9+10-11
6b	Any changes to trial outcomes after the trial commenced, with reasons		na
Sample size:			
7a	How sample size was determined	Method of calculation, number of clusters(s) (and whether equal or unequal cluster sizes are assumed), cluster size, a coefficient of intracluster correlation (ICC or *k*), and an indication of its uncertainty	10
7b	When applicable, explanation of any interim analyses and stopping guidelines		na
**Randomisation**			
Sequence generation:			
8a	Method used to generate the random allocation sequence		8
8b	Type of randomisation; details of any restriction (such as blocking and block size)	Details of stratification or matching if used	6-7

© 2025 Gude MF et al. *JAMA Network Open*.

© 2025 Gude MF et al. *JAMA Network Open*.

Section/topic and item No	Standard checklist item	Extension for cluster designs	Page No*
Allocation concealment mechanism:			
9	Mechanism used to implement the random allocation sequence (such as sequentially numbered containers), describing any steps taken to conceal the sequence until interventions were assigned	Specification that allocation was based on clusters rather than individuals and whether allocation concealment (if any) was at the cluster level, the individual participant level, or both	9
Implementation:			
10	Who generated the random allocation sequence, who enrolled participants, and who assigned participants to interventions	Replaced by 10a, 10b, and 10c	9
10a		Who generated the random allocation sequence, who enrolled clusters, and who assigned clusters to interventions	6-8
10b		Mechanism by which individual participants were included in clusters for the purposes of the trial (such as complete enumeration, random sampling)	6-8
10c		From whom consent was sought (representatives of the cluster, or individual cluster members, or both) and whether consent was sought before or after randomisation	na + Suppl material
Blinding:			
11a	If done, who was blinded after assignment to interventions (for example, participants, care providers, those assessing outcomes) and how		8
11b	If relevant, description of the similarity of interventions		na
Statistical methods:			
12a	Statistical methods used to compare groups for primary and secondary outcomes	How clustering was taken into account	10-11
12b	Methods for additional analyses, such as subgroup analyses and adjusted analyses		10-11+eTable 3
**Results**			
Participant flow (a diagram is strongly recommended):			Figure 1 + eFigure1
13a	For each group, the numbers of participants who were randomly assigned, received intended treatment, and were analysed for the primary outcome	For each group, the numbers of clusters that were randomly assigned, received intended treatment, and were analysed for the primary outcome	11-12+Fig 1+eFig 1+Tab 4
13b	For each group, losses and exclusions after randomisation, together with reasons	For each group, losses and exclusions for both clusters and individual cluster members	11-12+Tab 4
Recruitment:			
14a	Dates defining the periods of recruitment and follow-up		6-7
14b	Why the trial ended or was stopped		10
Baseline data:			
15	A table showing baseline demographic and clinical characteristics for each group	Baseline characteristics for the individual and cluster levels as applicable for each group	Tab 1+2+eTab 1
Numbers analysed:			
16	For each group, number of participants (denominator) included in each analysis and whether the analysis was by original assigned groups	For each group, number of clusters included in each analysis	11-12+Tab4 +eFigure 1
Outcomes and estimation:			

© 2025 Gude MF et al. *JAMA Network Open*.

Section/topic and item No	Standard checklist item	Extension for cluster designs	Page No*
17a	For each primary and secondary outcome, results for each group, and the estimated effect size and its precision (such as 95% confidence interval)	Results at the individual or cluster level as applicable and a coefficient of intracluster correlation (ICC or *k*) for each primary outcome	12-13 + tab 4
17b	For binary outcomes, presentation of both absolute and relative effect sizes is recommended		12-13 + tab 4
Ancillary analyses:			
18	Results of any other analyses performed, including subgroup analyses and adjusted analyses, distinguishing prespecified from exploratory		13+ eTab 3
Harms:			
19	All important harms or unintended effects in each group (for specific guidance see CONSORT for harms106)		13 + Tab 4
**Discussion**			
Limitations:			
20	Trial limitations, addressing sources of potential bias, imprecision, and, if relevant, multiplicity of analyses		15-16
Generalisability:			
21	Generalisability (external validity, applicability) of the trial findings	Generalisability to clusters and/or individual participants (as relevant)	15-16
Interpretation:			
22	Interpretation consistent with results, balancing benefits and harms, and considering other relevant evidence		14-16
**Other information**			
Registration:			
23	Registration number and name of trial registry		4+11
Protocol:			
24	Where the full trial protocol can be accessed, if available		Suppl Material
Funding:			
25	Sources of funding and other support (such as supply of drugs), role of funders		17

© 2025 Gude MF et al. *JAMA Network Open*.

eAppendix 2. CAM-VISION trial data availability confirmation

Regionshuset
Viborg

Region Midtjylland
Skottenborg 26
Postboks 21
DK-8800 Viborg
Tel. +45 7841 0000
kontakt@rm.dk
www.rm.dk

To whom it may concern,

Dato 9-12-2024
Sagsbehandler Maja Würgler Hansen
MAWHAN@rm.dk
+4524420260
Sagsnr. 1-45-70-55-23

The Legal Office of the Central Denmark Region hereby confirms that permission to pass on data for the CAM-VISION trial was granted on August 28, 2023.

This approval is documented in the Danish file titled: 'Godkendelse af ansøgning om videregivelse af oplysninger fra patientjournaler til brug for forskningsprojekt: "Dispatch of emergency call using video streaming compared with traditional telephone communication (CAM-VISION trial)"' with case number 1-45-70-55-23.

The data authorized for transmission includes both in-hospital and prehospital sources. In accordance with Danish legislation (The Danish Health Act § 46, 2) such data cannot be accessed or utilized for research purposes without prior approval.

1

Best regards,

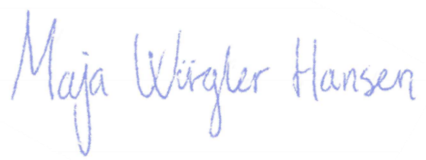

Maja Würgler Hansen
Legal consultant

© 2025 Gude MF et al. *JAMA Network Open*.
