# Supplementary material for: Video Streaming or Telephone Communication During Emergency Medical Services Dispatch Calls: A Cluster Randomized Clinical Trial
Source: JAMA Netw Open. 2025 Jul 1;8(7):e2519020. doi: 10.1001/jamanetworkopen.2025.19020 (PMC12215568; doi:10.1001/jamanetworkopen.2025.19020)
Supplement: Supplement 2. — Trial Protocol [file jamanetwopen-e2519020-s002.pdf]

Supplemental material – Protocols and SAP

Table of Content

PROTOCOL version 1.0 2
PROTOCOL version 2.0 (latest).. 20
Changes from protocol version 1.0 to 2.0 41
Statistical Analysis Plan (SAP) 1.0.. 42

PROTOCOL version 1.0

Protocol authors: Milena Meisner-Jensen & Martin Faurholdt Gude

THE CAM-VISION TRIAL

(Criteria based Ambulance Management – Video Indexation VS Standard Indexation On Non-selected emergency calls)

Principal Investigator Name: Martin Faurholdt Gude

Protocol: v1, 02 May 2022

Background

The Central Denmark Region's prehospital emergency service is currently enhancing its operations by incorporating live video streaming from callers to emergency medical service (EMS) dispatchers. This innovative approach aims to substantially improve the accuracy of dispatch decisions, particularly in determining the required urgency level.

Annually, the Emergency Medical Dispatch Center (EMDC) in the Central Denmark Region receives approximately 58,000 emergency calls.(1) In Denmark, every emergency call is answered by a nurse or paramedic who is specifically trained in dispatching emergencies. The EMS dispatcher decides on the EMS response, including the type and number of resources to allocate, such as an ambulance, physician-manned vehicle, and/or physician-manned helicopter (HEMS), as well as the urgency level. This decision-making process is supported by a standardized national criteria-based dispatch tool, the Danish Index for Emergency Care.(2)

This dispatch tool categorizes the patient's primary concern into distinct symptom groups (chapters), facilitating the assessment of the required urgency level and the appropriate allocation of resources. (3)

EMS dispatchers face the challenge of making critical decisions under stringent time constraints, as an A-response, indicating the highest urgency, must be initiated within just 2 minutes. Additionally, dispatchers rely solely on auditory information, which can sometimes hinder their understanding of complex clinical scenarios. Introducing video streaming to visualize the clinical presentation has been proposed to enhance and strengthen the decision-making process for EMS dispatchers.(4–7)

Video streaming is currently utilized in out-of-hours primary care in Denmark. In spring 2022, the technology became available to the EMDC Central Denmark Region and was tested in a three-month pilot project. The results from the pilot project revealed that three EMS dispatchers used video streaming in approximately 15% of their calls (data not shown). The pilot project also indicated that video streaming influenced the allocated level of urgency, consistent with data from another EMDC.(8) However, a drawback of video streaming is the increased duration of individual calls.(8,9) Following the pilot project, the subsequent implementation of video streaming in the EMDC is organized to adhere to this cluster randomized CAM-VISION trial.

Previous research has examined simulation trials focusing on the technology and feasibility of video streaming in emergency medical settings.(10) Other studies have retrospectively assessed the use of video streaming to improve care for specific medical issues, such as cardiac arrest.(11,12) One study reported that video streaming was attempted in only 1.4% of all emergency calls to the EMDC.(8)

To our knowledge, no ongoing or completed studies have evaluated the use of video streaming for all emergency calls with a randomized setup for comparison to the usual telephone-only (audio-only) communication.

The CAM-VISION trial aims to compare the proportion of allocated A-responses (highest urgency) when using video streaming versus telephone-only (audio-only) communication. Additionally, the trial aims to explore adverse consequences by examining mortality, ICU admission rates, hospital length-of-stay, and readmission rates within 24 hours after decisions on non-conveyance. Furthermore, it seeks to examine prehospital management in terms of precise dispatching.

Objectives

This study aims to compare emergency dispatches utilizing video streaming with traditional telephone-based (audio-only) communication for emergency calls at the EMDC Central Denmark Region. The evaluation will focus on the allocated level of urgency, allocated resources, and the Danish index chapters used for dispatching to assess the accuracy of EMS dispatchers in identifying specific conditions. Additionally, adverse events will be examined by determining mortality rates according to the level of urgency, hospital length of stay, and admission to intensive care units (ICU).

Given the complexity of integrating a new tool such as video streaming, which affects not only response allocation but also EMS dispatchers' perceptions of their work, two additional qualitative studies will explore the implementation process and the perceptions of both EMS dispatchers and citizens (not described in this protocol).

Primary Objective

To evaluate the frequency of dispatches with the highest level of urgency (A-responses).

Secondary Objectives

To assess the frequency of dispatches across all levels of urgency (A, B, C, D, and E).

To determine:

- The 30-day mortality rate following study inclusion,
- The consistency of urgency levels assigned to participants by the ambulance service when traveling to and from the scene,
- The length-of-hospital-stay for participants,
- The number of participants requiring ICU admission,
- The number of emergency calls where the dispatched level of urgency is changed during the call,
- The frequency of emergency calls where the initially dispatched level of urgency is subsequently lowered,
- The frequency of emergency calls where the initially dispatched level of urgency is subsequently increased,

- The number of emergency calls where the allocated resources are changed during the call,
- The number of participants readmitted to the hospital within 24 hours after a dispatch without hospital admission (response level E),
- The duration of emergency medical calls,
- The time from emergency call to dispatch and
- The on-scene time for emergency responses.

Other Objectives

To determine:

- The number of participants with return-of-spontaneous circulation (ROSC) after cardiac arrest,
- The 90-day neurological outcome after cardiac arrest using the cerebral performance scale (CPC),
- The 90-day functional outcome after cardiac arrest using the modified Rankin Scale (mRS),
- The number of patients with Stroke or TIA identified by EMS dispatchers,
- The rate of revascularization treatment (intravenous thrombolysis and/or endovascular treatment (EVT)) among patients with acute ischemic stroke,
- The rate of primary admission to hospitals offering acute stroke treatment,
- The onset-to-treatment times for patients with acute ischemic stroke treated with intravenous thrombolysis and onset-to-groin-puncture for patients treated with EVT,
- The number of lowest level of urgency (response level E) dispatches among children (< 15 years of age),
- The re-admissions among children (< 15 years of age) to the hospital within 24 hours from a prior emergency call dispatched with the lowest level of urgency (response level E), and
- The number of dispatches for each level of urgency (response levels A, B, C, D, and E) among children (< 15 years of age)

Hypothesis

Primary Hypothesis

The integration of video streaming in emergency medical dispatch communication, compared to telephone-only communication, will result in a reduced proportion of dispatches classified as A-responses, indicating the highest level of urgency.

Secondary Hypotheses

1. Urgency Level Correlation: Video streaming will increase the proportion of cases where the level of urgency assigned by the EMS dispatcher for scene arrival correspond the level of urgency assigned by the EMS provider for scene departure to the hospital.
2. Response Level Stability: There will be a reduction in the proportion of dispatches where the response level and/or allocated resources are altered during the emergency call.
3. No Resources Allocated: Video streaming will increase the proportion of response level E cases (indicating no allocated resources).
4. 30-Day Mortality Rates: There will be no change in the 30-day mortality rates for patients categorized based on the EMDC response level.
5. Call Duration: The median total call duration will increase, but the time from the call to dispatch will increase by less than 2 minutes.

Enhanced recognition by EMS dispatchers of specific conditions, specifically:

- Stroke and transient ischemic attack (TIA) cases
- Pediatric cases not warranting acute hospital admission (response level E), with no subsequent readmission within 24 hours and without an elevation in mortality among children classified under response level E.
- Instances of bystander-performed cardio-pulmonary resuscitation (CPR) characterized by low quality, thereby potentially improving the chances of achieving Return of Spontaneous Circulation (ROSC) upon correction.

Through these hypotheses, we seek to explore the potential benefits and enhancements that video streaming could introduce to the emergency medical dispatch process.

Method

Trial Design

This prospective, single-center cluster randomized trial (CRT) employs a parallel two-arm design with one cluster per arm. The staggered implementation ensures controlled and systematic deployment.

CRTs are particularly effective for evaluating interventions on entire populations, especially when logistical or organizational factors might influence the control group.(13) This trial investigates an educational intervention among EMS dispatchers. Individual patient-level randomization is impractical because once EMS dispatchers learn to integrate video streaming into their dispatch process, they might intentionally use these skills during telephone-only emergency calls for patient safety. This potential contamination of the control group underscores the necessity of a cluster design. Additionally, the urgent nature of EMS dispatch scenarios precludes obtaining informed consent from patients, further justifying the use of cluster randomization.

EMS dispatchers will be divided into two equally sized clusters: one using video streaming and the other using traditional telephone-only (audio-only) communication. Members of the video streaming cluster will receive training and are required to implement video streaming for all emergency calls. Although video streaming was initially optional, it is now mandatory for this cluster, which will be operational during the data collection phase. Video streaming should be initiated immediately after evaluating the patient's level of consciousness, before further categorization using the Danish Index, and prior to determining the appropriate response. In a few predefined cases requiring immediate action, video streaming will start after response allocation.

Study period

Due to organizational constraints on training capabilities, EMS dispatchers will be recruited in a staggered manner. Data collection will occur from January 1, 2023, to April 30, 2023.

Participants

The study involves EMS dispatchers from the EMDC in the Central Denmark Region. Over a four-month data collection period, all EMS dispatchers will be assigned to one of two groups: the control group (audio-only) or the intervention group (video streaming). Dispatchers who are not employed for the entire study period or who have not completed a six-week training period by the start date will be excluded. We expect to handle between 15,000 and 20,000 emergency calls during the trial.

Inclusion Criteria

- Emergency calls to EMS dispatchers in the EMDC,
- EMS dispatchers employed throughout the entire study period and
- EMS dispatchers who have completed a six-week training period by the start of the study.

Exclusion Criteria:

- Emergency calls directed to an EMDC physician or technical logistical personnel not using the criteria-based dispatch tool,
- Non-emergency calls, such as healthcare provider requested prehospital transports,
- EMS dispatchers not employed for the entire study period and
- EMS dispatchers who have not completed the six-week training period by the start of the study.

Arms and Interventions

Participant Groups and Interventions

Experimental: Intervention Group

Intervention Group:

Emergency Medical Services (EMS) dispatchers will use video-based communication during emergency calls.

Intervention Details:

EMS dispatchers will incorporate video streaming as an enhancement to the traditional audio-only communication with callers. This will be facilitated through DIAS (Dynamic Infrastructure for Applications and Services), a technologically platform developed by the Central Denmark Region specifically for patient consultations.

Device and Description:

The video streaming will be activated via a hyperlink sent to the caller's smartphone through a text message. This link allows the caller to initiate a live video feed using their smartphone camera, providing the dispatcher with a real-time visual of the emergency scene. The streaming connection is established directly between the dispatcher and the caller, ensuring a secure and immediate video link. Importantly, no data will be stored during or after the streaming session.

This approach aims to enhance the dispatchers' ability to assess and manage emergency situations by supplementing traditional audio communication with live video feed from the scene.

Active Comparator: Control Group

Control Group:

EMS dispatchers will use standard telephone communication (audio-only) during emergency calls.

Intervention Details:

EMS dispatchers will continue with the usual care protocol, which involves audio-only communication through standard telephone calls.

Device and Description:

Communication will be maintained exclusively via the telephone microphone. This control group will not receive training in video streaming, ensuring that the standard protocol of audio-only communication is followed during emergency medical dispatch, without incorporating video streaming.

This control group will allow for a comparison of the effectiveness between the traditional audio-only communication and the enhanced video-based communication used in the intervention group.

Patients

All patients or bystanders who initiates a contact to the healthcare system by an emergency call. Because of the equal sized clusters of EMS dispatcher, the emergency calls will be performed with or without the use of video streaming in a random order.

Randomization Process

The EMDC in the Central Denmark Region employs approximately 25 EMS dispatchers at any given time. These dispatchers are randomly assigned to one of two equally sized groups: the intervention group, which utilizes video streaming, and the control group, which uses traditional audio-only communication. All emergency calls are handled using either video streaming or audio-only communication, based on the dispatcher's assigned group. It is anticipated that the control group may handle a slightly higher volume of calls due to the additional time needed to set up and manage video streaming.

Matched Design for Cluster Randomization

Due to the small sample size and diverse backgrounds of the EMS providers, cluster randomization is performed using a matched design to minimize bias. The EMS dispatchers are matched based on their pre-study

tendencies to dispatch response level A, their average call time, and their years of experience. The matching prioritizes the proportion of A-responses dispatched over a 3-month period (January 1 - March 31, 2022), as the rate of A-responses is the primary outcome measure.

Matching Procedure

1. Initial Matching: Dispatchers with similar tendencies to dispatch A-responses are paired.
2. Secondary Matching: When multiple pairs have similar A-response tendencies, they are further matched based on call time (categorized into <33rd percentile, 33rd-66th percentile, or >66th percentile) and years of experience (categorized similarly).
3. Random Assignment: One dispatcher from each pair is randomly assigned to the video streaming cluster, while the other is assigned to the control cluster.

Implementation and Training

Following the randomization, EMS providers in the video streaming cluster undergo training, including individual feedback and support from the research team. EMS providers ready for implementation by January 1, 2023, join the intervention cluster for the entire study period, with their counterparts remaining in the control cluster. To maintain operational stability, EMS providers not ready by January 1 are excluded from the study.

Anonymity and Blinding

The identities of the EMS dispatchers are anonymized, and the researchers performing the matching and cluster allocation are blinded to these identities. This ensures unbiased and fair distribution of the EMS dispatchers between the intervention and control groups.

Outcomes

Primary outcomes

- A-responses (highest urgency) and non-A-responses.

Secondary outcomes

- All response levels (A, B, C and E responses).
- Mortality (30 days) in the total population and in response level groups.
- Number of matching response levels between the response level dispatched from the EMDC (ambulance transport to the scene) and the response level determined by the EMS providers (ambulance transport to hospital).
- Length-of-stay at hospital.
- Rate of admission to ICU.
- Number of emergency calls where the EMS dispatchers change level of response during the call.
- Number of dispatched response level E and the proportion of readmission within 24 hours.
- Length of emergency call.
- Length of the dispatch time (time from emergency call to dispatch).
- On-scene time (from ambulance arrival to departure).

Sub study outcomes

Data from the CAM-VISION trial will be used for sub studies including the following outcomes:

- Cardiac arrest: Return-of-spontaneous circulation (ROSC) after cardiac arrest.
- Cardiac arrest: Neurological outcome after surviving cardiac arrest.
- Stroke: Recognition of stroke or transient ischemic attack (TIA) based on the Danish Index (chapter 26 and the subheadings A03, A04 and B06).
- Stroke: Rate of acute stroke treatment (i.v. thrombolysis and/or endovascular treatment (EVT)) among patients with a timely contact to the EMDC (within 3 hours and 24 hours).

- Stroke: Primary admission to a hospital offering acute stroke treatment.
- Stroke: Onset-to-treatment times for patients treated with i.v. thrombolysis and onset-to-groin-puncture for patients treated with EVT.
- Children: Number and proportion of patients not admitted to hospital (non-conveyance) - response level E.
- Children: Re-admissions within 24 hours from emergency call to the EMDC.
- Children: All response levels (A, B, E).

Statistical considerations and analysis

Intention to Treat (Video Stream)

The intention-to-treat (ITT) population for the video stream study includes all patients allocated to either exposure group.

The following statistical methods will be employed to analyze the data:

Binary Outcomes:

- Risk Differences (RD): Analyzed using linear regression.
- Relative Risks (RR): Analyzed using Poisson regression.
- Both analyses will apply robust variance estimation.

Continuous Outcomes:

- General Analysis: Conducted using linear regression.
- Time Variables (Right Censored): Analyzed using Tobit regression.

Time-to-Event Variables:

- Analyzed using Cox regression analysis.

Ordered Outcomes (CPC and mRS):

- Analyzed using ordered logistic regression.

- Evaluated using the concordance index.

Cluster Randomization

To account for cluster randomization, cluster-robust variance will be implemented in all regression analyses. A unique cluster ID will be assigned to each EMS dispatcher.

Effect Measures

All effect measures will be presented with 95% confidence intervals. All analyses will be conducted using Stata 17 software. No per-protocol analyses will be performed due to the substantial risk of selection bias.

Missing Data

- Anticipation and Handling: The anticipated connection between treatment allocation and loss to follow-up is expected to be minimal. Missing data, including outcomes, is assumed to be missing at random.
- Quantification: The extent of missing data will be presented for outcome variables to quantify its degree.

Summary of Study Data

- Structure: Summary tables will be structured with columns for each cluster.
- Descriptive Statistics:
    - Continuous variables will be summarized as mean with standard deviation or median with interquartile range (25th and 75th percentiles) based on non-missing data.
    - Categorical and binary measures will be summarized by frequency and percentages, calculated based on the non-missing sample size.

Data Sources & Management

Upon conclusion of the trial period, data will be collected from the following sources:

1. Logis

Database description: The Logis database is a computer-aided dispatch system used by the Emergency Medical Dispatch Center (EMDC). It serves as a comprehensive repository that facilitates the efficient management of prehospital emergency services.

Data description:

- **Voice Logs:** Records all communication between dispatchers and emergency responders.
- **Assigned Indexations:** Categorizes emergencies based on the Danish index, including main complaint, dispatched level of urgency, and resources allocated.
- **Timestamps:** Captures detailed timestamps of various stages of prehospital management, including:
  - Time of the emergency call
  - Ambulance dispatch time
  - Arrival time at the scene
  - Departure time from the scene
  - Arrival time at the hospital

2. REDCap (Research Electronic Data Capture)

Database description: REDCap is a secure, web-based platform designed for the collection and management of research data.

Data description:

  - Data Collected: Within the trial, all calls utilizing video streaming will be recorded by EMS dispatchers using REDCap, ensuring real-time data entry, secure storage, and comprehensive audit trails for data integrity and compliance.

2. Prehospital Patient Record (ePPR)

Database description: The electronic Prehospital Patient Record (ePPR) stores comprehensive patient data within prehospital emergency services, encompassing information from ambulances, physician-manned vehicles, and helicopters from the Helicopter Emergency Medical Service (HEMS).

Data description:

- **Patient Information:** Includes age, sex, and addresses.
- **Vital parameter:** Records blood pressure, pulse, oxygen saturation, respiratory rate, Glasgow Coma Scale (GCS), neurological status, temperature, and other critical metrics.
- **Treatments Administered:** Details interventions such as Heart-Lung-Resuscitation (HLR), administration of medications (e.g., anticoagulants, tranexamic acid), and use of fluids, blood products, plasma, and medical equipment.
- **Point-of-care tests:** Includes tests like troponin T (TnT) for suspected acute coronary syndrome, arterial blood gas analysis, and blood glucose levels.
- **Operational Details:** Allocates urgency based on EMT/paramedic assessments on-site, and records time intervals and on-site duration.

Electronic Hospital Patient Record (eHPR)

Database description: The electronic Hospital Patient Record (eHPR) is a comprehensive repository that stores all patient data related to hospitalization, generated by healthcare personnel in hospitals.

Data description:

- **Admission Data:** Captures details of admission and re-admission, length of hospital stay, ICU admissions, and duration.
- **Mortality:** Records time and date of death.
- **Diagnosis and Treatments:** Documents final diagnosis (ICD-10) including co-diagnoses, procedure codes, and medication administration.

- **Medical History:** Includes information on comorbidities from prior ICD-10 registrations, enabling the generation of a 10-year comorbidity profile.
- **Measurements and Laboratory Tests:** Records vital parameters and lab tests conducted during the hospital stay.

Data Integration and Cross-Linking

Data from these sources will be cross-linked via the individual patient's social security number (CPR-number). This allows for comprehensive tracking of a patient requiring pre-hospital emergency services, from the initial call through ambulance/emergency physician intervention to the final diagnosis.

Permissions and Access

Relevant permissions for access to and use of data will be applied for to ensure compliance with ethical and legal standards.

Ethics

The implementation of video streaming was decided by the management of the pre-hospital emergency services in the Central Denmark Region as a tool to improve dispatch quality, independent of the cluster randomized trial. Since video streaming serves as an additional decision-making aid within regular operations and is not dictated by the study, no application for ethical approval is needed. A statement from the local ethical committee will only be sought if required by ClinicalTrials.gov during registration.

Dissemination

The trial will be registered at ClinicalTrials.gov. The study's findings will be published in an international peer-reviewed journal, regardless of whether the results are negative, positive, or inconclusive. Additionally, the results will be presented at both national and international conferences.

References

1. Lindskou TA, Mikkelsen S, Christensen EF, Hansen PA, Jørgensen G, Hendriksen OM, m.fl. The Danish prehospital emergency healthcare system and research possibilities. Scand J Trauma Resusc Emerg Med. 4. november 2019;27(1):100.

2. Emergency Medical Servises Denmark. Dansk Indeks for Akuthjælp (Danish Criteria Based Dispatch System for Emergencies). https://www.ph.rm.dk/siteassets/prahospitalet/prahospitale-omrader/amk-vagtcentral/dansk-indeks-1.8---landsudgaven.pdf; 2017 jun.

3. Mikkelsen S, Lassen AT. The Danish prehospital system. Eur J Emerg Med. december 2020;27(6):394–5.

4. Linderoth G, Hallas P, Lippert FK, Wibrandt I, Loumann S, Møller TP, m.fl. Challenges in out-of-hospital cardiac arrest - A study combining closed-circuit television (CCTV) and medical emergency calls. Resuscitation. november 2015;96:317–22.

5. Linderoth G, Møller TP, Folke F, Lippert FK, Østergaard D. Medical dispatchers' perception of visual information in real out-of-hospital cardiac arrest: a qualitative interview study. Scand J Trauma Resusc Emerg Med. 25. januar 2019;27(1):8.

6. Clay-Williams R, Baysari M, Taylor N, Zalitis D, Georgiou A, Robinson M, m.fl. Service provider perceptions of transitioning from audio to video capability in a telehealth system: a qualitative evaluation. BMC Health Serv Res. 14. august 2017;17(1):558.

7. Lin YY, Chiang WC, Hsieh MJ, Sun JT, Chang YC, Ma MHM. Quality of audio-assisted versus video-assisted dispatcher-instructed bystander cardiopulmonary resuscitation: A systematic review and meta-analysis. Resuscitation. februar 2018;123:77–85.

8. Linderoth G, Lippert F, Østergaard D, Ersbøll AK, Meyhoff CS, Folke F, m.fl. Live video from bystanders' smartphones to medical dispatchers in real emergencies. BMC Emerg Med. 6. september 2021;21(1):101.

9. Cicero MX, Walsh B, Solad Y, Whitfill T, Paesano G, Kim K, m.fl. Do you see what I see? Insights from using google glass for disaster telemedicine triage. Prehospital Disaster Med. februar 2015;30(1):4–8.

10. Ter Avest E, Lambert E, de Coverly R, Tucker H, Griggs J, Wilson MH, m.fl. Live video footage from scene to aid helicopter emergency medical service dispatch: a feasibility study. Scand J Trauma Resusc Emerg Med. 8. maj 2019;27(1):55.

11. Lee SY, Song KJ, Shin SD, Hong KJ, Kim TH. Comparison of the effects of audio-instructed and video-instructed dispatcher-assisted cardiopulmonary resuscitation on resuscitation outcomes after out-of-hospital cardiac arrest. Resuscitation. 1. februar 2020;147:12–20.

12. Lee HS, You K, Jeon JP, Kim C, Kim S. The effect of video-instructed versus audio-instructed dispatcher-assisted cardiopulmonary resuscitation on patient outcomes following out of hospital cardiac arrest in Seoul. Sci Rep. 30. juli 2021;11:15555.

13. Hurley JC. How the Cluster-randomized Trial "Works". Clin Infect Dis Off Publ Infect Dis Soc Am. 2. januar 2020;70(2):341–6.

PROTOCOL version 2.0 (latest)

Authors of protocol changes: Natascha H Bohnstedt-Pedersen & Martin Faurholdt Gude

THE CAM-VISION TRIAL

(Criteria based Ambulance Management – Video Indexation VS Standard Indexation On Non-selected emergency calls)

ClinicalTrials.gov Identifier: NCT05742412

Principal Investigator Name: Martin Faurholdt Gude

Protocol: v2, 23 February 2023

Background

The Central Denmark Region's prehospital emergency service is currently enhancing its operations by incorporating live video streaming from callers to emergency medical service (EMS) dispatchers. This innovative approach aims to substantially improve the accuracy of dispatch decisions, particularly in determining the required urgency level.

Annually, the Emergency Medical Dispatch Center (EMDC) in the Central Denmark Region receives approximately 58,000 emergency calls.(1) In Denmark, every emergency call is answered by a nurse or paramedic who is specifically trained in dispatching emergencies. The EMS dispatcher decides on the EMS response, including the type and number of resources to allocate, such as an ambulance, physician-manned vehicle, and/or physician-manned helicopter (HEMS), as well as the urgency level. This decision-making process is supported by a standardized national criteria-based dispatch tool, the Danish Index for Emergency Care.(2)

This dispatch tool categorizes the patient's primary concern into distinct symptom groups (chapters), facilitating the assessment of the required urgency level and the appropriate allocation of resources. (3)

EMS dispatchers face the challenge of making critical decisions under stringent time constraints, as an A-response, indicating the highest urgency, must be initiated within just 2 minutes. Additionally, dispatchers rely solely on auditory information, which can sometimes hinder their understanding of complex clinical scenarios. Introducing video streaming to visualize the clinical presentation has been proposed to enhance and strengthen the decision-making process for EMS dispatchers.(4–7)

Video streaming is currently utilized in out-of-hours primary care in Denmark. In spring 2022, the technology became available to the EMDC Central Denmark Region and was tested in a three-month pilot project. The results from the pilot project revealed that three EMS dispatchers used video streaming in approximately 15% of their calls (data not shown). The pilot project also indicated that video streaming influenced the allocated level of urgency, consistent with data from another EMDC.(8) However, a drawback of video streaming is the increased duration of individual calls.(8,9) Following the pilot project, the subsequent implementation of video streaming in the EMDC is organized to adhere to this cluster randomized CAM-VISION trial.

Previous research has examined simulation trials focusing on the technology and feasibility of video streaming in emergency medical settings.(10) Other studies have retrospectively assessed the use of video streaming to improve care for specific medical issues, such as cardiac arrest.(11,12) One study reported that video streaming was attempted in only 1.4% of all emergency calls to the EMDC.(8)

To our knowledge, no ongoing or completed studies have evaluated the use of video streaming for all emergency calls with a randomized setup for comparison to the usual telephone-only communication.

The CAM-VISION trial aims to compare the proportion of allocated A-responses (highest urgency) when using video streaming versus telephone-only communication. Additionally, the trial aims to explore adverse consequences by examining mortality, ICU admission rates, hospital length-of-stay, and readmission rates within 24 hours after decisions on non-conveyance. Furthermore, it seeks to examine prehospital management in terms of precise dispatching.

Objectives

This study aims to compare emergency dispatches utilizing video streaming with traditional telephone-based communication for emergency calls at the EMDC Central Denmark Region. The evaluation will focus on the allocated level of urgency, allocated resources, and the Danish index chapters used for dispatching to assess the accuracy of EMS dispatchers in identifying specific conditions. Additionally, adverse events will be examined by determining mortality rates according to the level of urgency, hospital length of stay, and admission to intensive care units (ICU).

Given the complexity of integrating a new tool such as video streaming, which affects not only response allocation but also EMS dispatchers' perceptions of their work, two additional qualitative studies will explore the implementation process and the perceptions of both EMS dispatchers and citizens (not described further in this protocol).

Primary Objective

To evaluate the frequency of dispatches with the highest level of urgency (A-responses).

Secondary Objectives

To assess the frequency of dispatches across all levels of urgency (A, B, C, D, and E).

To determine:

- The 30-day mortality rate following study inclusion,
- The consistency of urgency levels assigned to participants by the ambulance service when traveling to and from the scene,
- The length-of-hospital-stay for participants,
- The number of participants requiring ICU admission,
- The number of emergency calls where the dispatched level of urgency is changed during the call,
- The frequency of emergency calls where the initially dispatched level of urgency is subsequently lowered,
- The frequency of emergency calls where the initially dispatched level of urgency is subsequently increased,

- The number of emergency calls where the allocated resources are changed during the call,
- The number of participants readmitted to the hospital within 24 hours after a dispatch without hospital admission (response level E),
- The duration of emergency medical calls,
- The time from emergency call to dispatch and
- The on-scene time for emergency responses.

Other Objectives

To determine:

- The number of participants with return-of-spontaneous circulation (ROSC) after cardiac arrest,
- The 90-day neurological outcome after cardiac arrest using the cerebral performance scale (CPC),
- The 90-day functional outcome after cardiac arrest using the modified Rankin Scale (mRS),
- The number of patients with Stroke or TIA identified by EMS dispatchers,
- The rate of revascularization treatment (intravenous thrombolysis and/or endovascular treatment (EVT)) among patients with acute ischemic stroke,
- The rate of primary admission to hospitals offering acute stroke treatment,
- The onset-to-treatment times for patients with acute ischemic stroke treated with intravenous thrombolysis and onset-to-groin-puncture for patients treated with EVT,
- The number of lowest level of urgency (response level E) dispatches among children (< 15 years of age),
- The re-admissions among children (< 15 years of age) to the hospital within 24 hours from a prior emergency call dispatched with the lowest level of urgency (response level E), and
- The number of dispatches for each level of urgency (response levels A, B, C, D, and E) among children (< 15 years of age)

Hypothesis

Primary Hypothesis

The integration of video streaming in emergency medical dispatch communication, compared to telephone-only communication, will result in a reduced proportion of dispatches classified as A-responses, indicating the highest level of urgency.

Secondary Hypotheses

6. Urgency Level Correlation: Video streaming will increase the proportion of cases where the level of urgency assigned by the EMS dispatcher for scene arrival correspond the level of urgency assigned by the EMS provider for scene departure to the hospital.
7. Response Level Stability: There will be a reduction in the proportion of dispatches where the response level and/or allocated resources are altered during the emergency call.
8. No Resources Allocated: Video streaming will increase the proportion of response level E cases (indicating no allocated resources).
9. 30-Day Mortality Rates: There will be no change in the 30-day mortality rates for patients categorized based on the EMDC response level.
10. Call Duration: The median total call duration will increase, but the time from the call to dispatch will increase by less than 2 minutes.

Enhanced recognition by EMS dispatchers of specific conditions, specifically:

- Stroke and transient ischemic attack (TIA) cases
- Pediatric cases not warranting acute hospital admission (response level E), with no subsequent readmission within 24 hours and without an elevation in mortality among children classified under response level E.
- Instances of bystander-performed cardio-pulmonary resuscitation (CPR) characterized by low quality, thereby potentially improving the chances of achieving Return of Spontaneous Circulation (ROSC) upon correction.

Through these hypotheses, we seek to explore the potential benefits and enhancements that video streaming could introduce to the emergency medical dispatch process.

Method

Trial Design

This prospective, single-center cluster randomized trial (CRT) employs a parallel two-arm design with one cluster per arm. The staggered implementation ensures controlled and systematic deployment.

CRTs are particularly effective for evaluating interventions on entire populations, especially when logistical or organizational factors might influence the control group.(13) This trial investigates an educational intervention among EMS dispatchers. Individual patient-level randomization is impractical because once EMS dispatchers learn to integrate video streaming into their dispatch process, they might intentionally use these skills during telephone-only emergency calls for patient safety. This potential contamination of the control group underscores the necessity of a cluster design. Additionally, the urgent nature of EMS dispatch scenarios precludes obtaining informed consent from patients, further justifying the use of cluster randomization.

EMS dispatchers will be divided into two equally sized clusters: one using video streaming and the other using traditional telephone-only communication. Members of the video streaming cluster will receive training and are required to implement video streaming for all emergency calls. Although video streaming was initially optional, it is now mandatory for this cluster, which will be operational during the data collection phase.

Video streaming should be initiated immediately after evaluating the patient's level of consciousness, before further categorization using the Danish Index, and prior to determining the appropriate response. In a few predefined cases requiring immediate action, video streaming will start after response allocation.

Study period

Due to organizational constraints on training capabilities, EMS dispatchers will be recruited in a staggered manner. Data collection will occur from January 1, 2023, to April 30, 2023.

Participants

The study involves EMS dispatchers from the EMDC in the Central Denmark Region. Over a four-month data collection period, all EMS dispatchers will be assigned to one of two groups: the control group (telephone-only) or the intervention group (video streaming). Dispatchers who are not employed throughout the study period or have not completed a six-week training program before the start date will be excluded. The trial is expected to process approximately 20,000 to 25,000 emergency calls, ensuring the inclusion of 15,000 to 20,000 calls in the study.

Inclusion Criteria

- Emergency calls to EMS dispatchers in the EMDC,
- EMS dispatchers employed throughout the entire study period and
- EMS dispatchers who have completed a six-week training period by the start of the study.

Exclusion Criteria:

- Emergency calls directed to an EMDC physician or technical logistical personnel not using the criteria-based dispatch tool,
- Non-emergency calls, such as healthcare provider requested prehospital transports,
- EMS dispatchers not employed for the entire study period and
- EMS dispatchers who have not completed the six-week training period by the start of the study.

Arms and Interventions

Participant Groups and Interventions

Experimental: Intervention Group

Intervention Group:

Emergency Medical Services (EMS) dispatchers will use video-based communication during emergency calls.

Intervention Details:

EMS dispatchers will incorporate video streaming as an enhancement to the traditional telephone-only communication with callers. This will be facilitated through DIAS (Dynamic Infrastructure for Applications and Services), a technologically platform developed by the Central Denmark Region specifically for patient consultations.

Device and Description:

The video streaming will be activated via a hyperlink sent to the caller's smartphone through a text message. This link allows the caller to initiate a live video feed using their smartphone camera, providing the dispatcher with a real-time visual of the emergency scene. The streaming connection is established directly between the dispatcher and the caller, ensuring a secure and immediate video link. Importantly, no data will be stored during or after the streaming session.

This approach aims to enhance the dispatchers' ability to assess and manage emergency situations by supplementing traditional telephone communication with live video feed from the scene.

Active Comparator: Control Group

Control Group:

EMS dispatchers will use standard telephone-only communication during emergency calls.

Intervention Details:

EMS dispatchers will continue with the usual care protocol, which involves standard telephone-only communication in medical emergency calls.

Device and Description:

Communication will be maintained exclusively via the telephone microphone. This control group will not receive training in video streaming, ensuring that the standard protocol of telephone-only communication is followed during emergency medical dispatch, without incorporating video streaming.

This control group will allow for a comparison of the effectiveness between the traditional telephone-only communication and the enhanced video-based communication used in the intervention group.

Patients

All patients or bystanders who initiates a contact to the healthcare system by an emergency call. Because of the equal sized clusters of EMS dispatcher, the emergency calls will be performed with or without the use of video streaming in a random order.

Randomization Process

The EMDC in the Central Denmark Region employs approximately 25 EMS dispatchers at any given time. These dispatchers are randomly assigned to one of two equally sized groups: the intervention group, which utilizes video streaming, and the control group, which uses traditional telephone-only communication. All emergency calls are handled using either video streaming or telephone-only communication, based on the dispatcher’s assigned group. It is anticipated that the control group may handle a slightly higher volume of calls due to the additional time needed to set up and manage video streaming.

Matched Design for Cluster Randomization

Due to the small sample size and diverse backgrounds of the EMS providers, cluster randomization is performed using a matched design to minimize bias. The EMS dispatchers are matched based on their pre-study

tendencies to dispatch response level A, their average call time, and their years of experience. The matching prioritizes the proportion of A-responses dispatched over a 3-month period (January 1 - March 31, 2022), as the rate of A-responses is the primary outcome measure.

Matching Procedure

4. Initial Matching: Dispatchers with similar tendencies to dispatch A-responses are paired.
5. Secondary Matching: When multiple pairs have similar A-response tendencies, they are further matched based on call time (categorized into <33rd percentile, 33rd-66th percentile, or >66th percentile) and years of experience (categorized similarly).
6. Random Assignment: One dispatcher from each pair is randomly assigned to the video streaming cluster, while the other is assigned to the control cluster.

Implementation and Training

Following the randomization, EMS providers in the video streaming cluster undergo training, including individual feedback and support from the research team. EMS providers ready for implementation by January 1, 2023, join the intervention cluster for the entire study period, with their counterparts remaining in the control cluster. To maintain operational stability, EMS providers not ready by January 1 are excluded from the study.

Anonymity and Blinding

The identities of the EMS dispatchers are anonymized, and the researchers performing the matching and cluster allocation are blinded to these identities. This ensures unbiased and fair distribution of the EMS dispatchers between the intervention and control groups.

Outcomes

Primary Outcome

1. **Frequency of Dispatches with the Highest Level of Urgency (A-Responses)**

- **Definition:** The frequency of dispatches with the highest level of urgency, as defined by dispatch codes generated by the computer-aided dispatch software (Logis) and stored in the EMDC database.
- **Timeframe:** Data will be collected continuously during the 4-month study period, with analysis conducted after study termination.

Secondary Outcomes

1. **Frequency of Dispatches Across All Levels of Urgency (A, B, C, D, and E)**
   - **Definition:** The frequency of dispatches across all levels of urgency, graded into five levels, as defined by the Logis system.
   - **Timeframe:** Data will be collected continuously during the study period, with analysis conducted post-study.
2. **30-Day Mortality**
   - **Definition:** Mortality measured 30 days after study inclusion.
   - **Timeframe:** Data will be collected 30 days after study termination at the earliest.
3. **Matching Response Levels Between EMS Dispatch and Providers**
   - **Definition:** The proportion of cases where the urgency level dispatched by the EMS dispatcher matches the level determined by EMS providers during ambulance transport to the hospital.
   - **Timeframe:** Data will be collected continuously during the study period, with analysis conducted post-study.
4. **Length of Stay in Hospital**
   - **Definition:** The time from initial hospital admission to discharge to home or a care facility.
   - **Timeframe:** Data will be collected 90 days post-study termination at the earliest.
5. **ICU Admissions**

- **Definition:** The number and proportion of patients requiring ICU admission during their hospital stay.
- **Timeframe:** Data will be collected continuously during the study period, with analysis conducted post-study.

6. **Emergency Calls with Changed Response Levels**
   - **Definition:** The frequency of emergency calls where the EMS dispatcher adjusts the level of urgency during the call.
   - **Timeframe:** Data will be collected continuously during the study period, with analysis conducted post-study.
7. **Emergency Calls with Resource Allocation Changes**
   - **Definition:** The frequency of calls where the initially allocated resources (e.g., ambulance, helicopter) are modified during the call.
   - **Timeframe:** Data will be collected continuously during the study period, with analysis conducted post-study.
8. **Readmissions Within 24 Hours After a Non-Admitted Call (Response Level E)**
   - **Definition:** The proportion of patients readmitted to the hospital within 24 hours of a call dispatched at the lowest urgency level (Response Level E), where no prehospital resources were allocated.
   - **Timeframe:** Data will be collected within 24 hours after study termination at the earliest.
9. **Duration of Emergency Calls**
   - **Definition:** The total time, in minutes, for each emergency call.
   - **Timeframe:** Data will be collected continuously during the study period, with analysis conducted post-study.

10. **Dispatch Time**
    - **Definition:** The time, in minutes, from the start of the emergency call to the dispatch of resources with a specified level of urgency.
    - **Timeframe:** Data will be collected continuously during the study period, with analysis conducted post-study.
11. **On-Scene Time**
    - **Definition:** The time, in minutes, from EMS arrival at the scene to departure with the patient.
    - **Timeframe:** Data will be collected continuously during the study period, with analysis conducted post-study.

Other Outcomes

Data from the CAM-VISION trial will be used for sub studies including the following outcomes:

1. **Cardiac Arrest**
    - **Definition:**
        - Return of Spontaneous Circulation (ROSC): The proportion of patients achieving sustained ROSC during the prehospital or in-hospital phase of treatment.
        - Neurological Outcomes: Favorable neurological function measured using CPC and mRS. CPC ranges from 1 (intact function) to 5 (brain death), and mRS ranges from 0 (no symptoms) to 6 (death). Favorable neurological outcomes are defined as CPC 1–2 or mRS 0–2.
    - **Timeframe:** Data will be collected up to 90 days post-study termination.
2. **Stroke and TIA**
    - **Definition:**

    - Recognition of stroke or transient ischemic attack (TIA) by the EMS dispatcher based on the Danish Index (chapter 26, subheadings A03, A04, and B06).
    - Rate of acute stroke treatments, including intravenous thrombolysis and/or EVT, among patients contacting the EMDC within three hours for thrombolysis and 24 hours for EVT.
    - Time from symptom onset to treatment, including onset-to-treatment for intravenous thrombolysis and onset-to-groin-puncture for EVT.
  - **Timeframe:** Data will be collected continuously during the study period, with analysis conducted post-study.

3. **Children (<15 Years)**
  - **Definition:**
    - Proportion of children not admitted to hospital after dispatch at the lowest level of urgency (Response Level E).
    - Readmissions of children to the hospital within 24 hours of an emergency call dispatched at the lowest level of urgency.
    - Distribution of response levels (A, B, C, D, and E) among children.
  - **Timeframe:** Data will be collected continuously during the study period, with analysis conducted post-study.

Statistical considerations and analysis

Intention to Treat (Video Stream)

The intention-to-treat (ITT) population for the video stream study includes all patients allocated to either exposure group.

The following statistical methods will be employed to analyze the data:

Binary Outcomes:

- Risk Differences (RD): Analyzed using linear regression.
- Relative Risks (RR): Analyzed using Poisson regression.
- Both analyses will apply robust variance estimation.

Continuous Outcomes:

- General Analysis: Conducted using linear regression.
- Time Variables (Right Censored): Analyzed using Tobit regression.

Time-to-Event Variables:

- Analyzed using Cox regression analysis.

Ordered Outcomes (CPC and mRS):

- Analyzed using ordered logistic regression.
- Evaluated using the concordance index.

Cluster Randomization

To account for cluster randomization, cluster-robust variance will be implemented in all regression analyses. A unique cluster ID will be assigned to each EMS dispatcher.

Effect Measures

All effect measures will be presented with 95% confidence intervals. All analyses will be conducted using Stata 17 software. No per-protocol analyses will be performed due to the substantial risk of selection bias.

Missing Data

- Anticipation and Handling: The anticipated connection between treatment allocation and loss to follow-up is expected to be minimal. Missing data, including outcomes, is assumed to be missing at random. In a sensitivity analysis missing will be handled using multiple imputations.
- Quantification: The extent of missing data will be presented for outcome variables to quantify its degree.

Summary of Study Data

- Structure: Summary tables will be structured with columns for each cluster.
- Descriptive Statistics:
    - Continuous variables will be summarized as mean with standard deviation or median with interquartile range (25th and 75th percentiles) based on non-missing data.
    - Categorical and binary measures will be summarized by frequency and percentages, calculated based on the non-missing sample size.

Data Sources & Management

Upon conclusion of the trial period, data will be collected from the following sources:

1. Logis

Database description: The Logis database is a computer-aided dispatch system used by the Emergency Medical Dispatch Center (EMDC). It serves as a comprehensive repository that facilitates the efficient management of prehospital emergency services.

Data description:

- **Assigned Indexations:** Categorizes emergencies based on the Danish index, including main complaint, dispatched level of urgency, and resources allocated.
- **Timestamps:** Captures detailed timestamps of various stages of prehospital management, including:
    - Time of the emergency call

- Ambulance dispatch time
- Arrival time at the scene
- Departure time from the scene
- Arrival time at the hospital

2. Telecommunication Database

Database description: The telecommunication database records all Emergency Medical Dispatch Center (EMDC) calls as voicelogs, serving as a repository for communication details during emergency calls.

Data description:

- **Voice Logs:** Records all communication between dispatchers and emergency responders., capturing the following details: EMS dispatcher ID; Call duration; Call date and time
- **Data Integration:** The telecommunication database is not directly linked to the Logis computer-aided dispatch (CAD) system.

3. REDCap (Research Electronic Data Capture)

Database description: REDCap is a secure, web-based platform designed for the collection and management of research data.

Data description:

- Data Collected: Within the trial, all calls utilizing video streaming will be recorded by EMS dispatchers using REDCap, ensuring real-time data entry, secure storage, and comprehensive audit trails for data integrity and compliance.

4. Prehospital Patient Record (ePPR)

Database description: The electronic Prehospital Patient Record (ePPR) stores comprehensive patient data within prehospital emergency services, encompassing information from ambulances, physician-manned vehicles, and helicopters from the Helicopter Emergency Medical Service (HEMS).

Data description:

- **Patient Information:** Includes age, sex, and addresses.
- **Vital parameter:** Records blood pressure, pulse, oxygen saturation, respiratory rate, Glasgow Coma Scale (GCS), neurological status, temperature, and other critical metrics.
- **Treatments Administered:** Details interventions such as Heart-Lung-Resuscitation (HLR), administration of medications (e.g., anticoagulants, tranexamic acid), and use of fluids, blood products, plasma, and medical equipment.
- **Point-of-care tests:** Includes tests like troponin T (TnT) for suspected acute coronary syndrome, arterial blood gas analysis, and blood glucose levels.
- **Operational Details:** Allocates urgency based on EMT/paramedic assessments on-site, and records time intervals and on-site duration.

5. Electronic Hospital Patient Record (eHPR)

Database description: The electronic Hospital Patient Record (eHPR) is a comprehensive repository that stores all patient data related to hospitalization, generated by healthcare personnel in hospitals.

Data description:

- **Admission Data:** Captures details of admission and re-admission, length of hospital stay, ICU admissions, and duration.
- **Mortality:** Records time and date of death.
- **Diagnosis and Treatments:** Documents final diagnosis (ICD-10) including co-diagnoses, procedure codes, and medication administration.

- **Medical History:** Includes information on comorbidities from prior ICD-10 registrations, enabling the generation of a 10-year comorbidity profile.
- **Measurements and Laboratory Tests:** Records vital parameters and lab tests conducted during the hospital stay.

Data Integration and Cross-Linking

Data from these sources will be cross-linked via the individual patient's social security number (CPR-number). This allows for comprehensive tracking of a patient requiring pre-hospital emergency services, from the initial call through ambulance/emergency physician intervention to the final diagnosis.

Permissions and Access

Relevant permissions for access to and use of data will be applied for to ensure compliance with ethical and legal standards.

Ethics

The implementation of video streaming was decided by the management of the pre-hospital emergency services in the Central Denmark Region as a tool to improve dispatch quality, independent of the cluster randomized trial. Since video streaming serves as an additional decision-making aid within regular operations and is not dictated by the study, no application for ethical approval is needed. A statement from the local ethical committee will only be sought if required by ClinicalTrials.gov during registration.

Dissemination

The trial will be registered at ClinicalTrials.gov. The study's findings will be published in an international peer-reviewed journal, regardless of whether the results are negative, positive, or inconclusive. Additionally, the results will be presented at both national and international conferences.

References

1. Lindskou TA, Mikkelsen S, Christensen EF, Hansen PA, Jørgensen G, Hendriksen OM, m.fl. The Danish prehospital emergency healthcare system and research possibilities. Scand J Trauma Resusc Emerg Med. 4. november 2019;27(1):100.

2. Emergency Medical Servises Denmark. Dansk Indeks for Akuthjælp (Danish Criteria Based Dispatch System for Emergencies). https://www.ph.rm.dk/siteassets/prahospitalet/prahospitale-omrader/amk-vagtcentral/dansk-indeks-1.8---landsudgaven.pdf; 2017 jun.

3. Mikkelsen S, Lassen AT. The Danish prehospital system. Eur J Emerg Med. december 2020;27(6):394–5.

4. Linderoth G, Hallas P, Lippert FK, Wibrandt I, Loumann S, Møller TP, m.fl. Challenges in out-of-hospital cardiac arrest - A study combining closed-circuit television (CCTV) and medical emergency calls. Resuscitation. november 2015;96:317–22.

5. Linderoth G, Møller TP, Folke F, Lippert FK, Østergaard D. Medical dispatchers' perception of visual information in real out-of-hospital cardiac arrest: a qualitative interview study. Scand J Trauma Resusc Emerg Med. 25. januar 2019;27(1):8.

6. Clay-Williams R, Baysari M, Taylor N, Zalitis D, Georgiou A, Robinson M, m.fl. Service provider perceptions of transitioning from audio to video capability in a telehealth system: a qualitative evaluation. BMC Health Serv Res. 14. august 2017;17(1):558.

7. Lin YY, Chiang WC, Hsieh MJ, Sun JT, Chang YC, Ma MHM. Quality of audio-assisted versus video-assisted dispatcher-instructed bystander cardiopulmonary resuscitation: A systematic review and meta-analysis. Resuscitation. februar 2018;123:77–85.

8. Linderoth G, Lippert F, Østergaard D, Ersbøll AK, Meyhoff CS, Folke F, m.fl. Live video from bystanders' smartphones to medical dispatchers in real emergencies. BMC Emerg Med. 6. september 2021;21(1):101.

9. Cicero MX, Walsh B, Solad Y, Whitfill T, Paesano G, Kim K, m.fl. Do you see what I see? Insights from using google glass for disaster telemedicine triage. Prehospital Disaster Med. februar 2015;30(1):4–8.

10. Ter Avest E, Lambert E, de Coverly R, Tucker H, Griggs J, Wilson MH, m.fl. Live video footage from scene to aid helicopter emergency medical service dispatch: a feasibility study. Scand J Trauma Resusc Emerg Med. 8. maj 2019;27(1):55.

11. Lee SY, Song KJ, Shin SD, Hong KJ, Kim TH. Comparison of the effects of audio-instructed and video-instructed dispatcher-assisted cardiopulmonary resuscitation on resuscitation outcomes after out-of-hospital cardiac arrest. Resuscitation. 1. februar 2020;147:12–20.

12. Lee HS, You K, Jeon JP, Kim C, Kim S. The effect of video-instructed versus audio-instructed dispatcher-assisted cardiopulmonary resuscitation on patient outcomes following out of hospital cardiac arrest in Seoul. Sci Rep. 30. juli 2021;11:15555.

13. Hurley JC. How the Cluster-randomized Trial “Works”. Clin Infect Dis Off Publ Infect Dis Soc Am. 2. januar 2020;70(2):341–6.

Changes from protocol version 1.0 to 2.0

Control Group

- **Specification of Name:** The control group name has been updated from "audio-only" to "telephone-only" for clarity and consistency.

Participants

- **Adjustment to Expected Emergency Call Volume:**

  The total volume was updated from "15,000 to 20,000" to "20,000 to 25,000," with an expected inclusion of 15,000 to 20,000 calls in the study. This adjustment accounts for the exclusion of EMS providers not employed throughout the study period or those who have not completed the required six-week training program prior to the study start.

Outcomes

- **Clarification and Detail:**

  The outcome section was updated to include definitions for each individual outcome along with their respective timeframes, ensuring greater transparency and alignment with study objectives.

Missing Data

- **Sensitivity Analysis Description:**

  The section on missing data was expanded to include a sensitivity analysis approach. In the sensitivity analysis, missing data will be handled using multiple imputations to ensure robustness in the analysis.

Data Sources and Management

- **Specification of Telecommunication Database:**

  The audio-recorded medical emergency call voicelogs were listed under the Logis database. This has been corrected as being stored in the telecommunication database.

Statistical Analysis Plan

TRIAL FULL TITLE	Dispatch of Emergency Call Using Video Streaming Compared With Traditional Telephone Communication (CAM-VISION)
Clinical Trial Identifier	NCT05742412
Unique Protocol ID	3436
SAP VERSION	1
SAP VERSION DATE	18.08.2023
TRIAL STATISTICIAN	Jan Brink Valentin
TRIAL PRINCIPAL INVESTIGATOR	Martin Faurholdt Gude
SAP AUTHOR(s)	Martin Faurholdt Gude & Jan Brink Valentin

1. SAP Signatures

I give my approval for the attached SAP.

Statistician

Name: Jan Brink Valentin

Signature: [signature]

Date: 2023-08-27

Principal Investigator

Name: Martin Faurholdt Gude

Signature: [signature]

Date: 27-08-2023

1.1 Table of Contents

1. SAP Signatures 1
1.1 Table of Contents 2
2. Study Objectives and Endpoints 2
  2.1 Study Objectives 2
  2.2. Study period 3
  2.3 Study setting and cluster design 3
  2.4 Endpoints 3
    Primary Outcome Measure 3
    Secondary Outcome Measures 3
    Other Pre-specified Outcome Measures: 4
3 Study Methods 5
  3.1 General Study Design and Plan 5
  3.2 Inclusion-Exclusion Criteria and General Study Population 5
  Inclusion criteria 5
  Exclusion criteria 5
  3.3 Randomization 6
  3.4 Sample Size 6
  3.5 Timing of Analyses 6
    SAP Signature 6
    Data Authorization 6
    Primary Analyses 6
    Secondary analyses 6
  3.6 Analysis Populations 6
    Intention to Treat (video stream) 6
  3.7 Missing Data 7
  3.8 Summary of Study Data 7

2. Study Objectives and Endpoints

2.1 Study Objectives

To investigate differences in allocated urgency level when emergency calls are dispatched by emergency medical service (EMS) utilizing video streaming as opposed to sole telephone (audio-only) communication.

Secondary aims include the association between the use of video streaming and secondary and exploratory endpoints listed below.

2.2. Study period

The study will be conducted from January 1, 2023, to April 30, 2023.

2.3 Study setting and cluster design

In the Central Denmark Region, a single Emergency Medical Dispatch Center (EMDC) handles all emergency calls (equivalent to 9-1-1 calls in the US). The region receives approximately 55,000 to 60,000 emergency calls annually and employs around 25 Emergency Medical Service (EMS) Dispatchers, all of whom are either nurses or paramedics. Within the EMDC, EMS dispatchers were divided into one of two clusters, each containing 10 EMS dispatchers. Due to the limited number of clusters, a matched-pair (MP) design was adopted. This design relied on specific criteria for matching, including the average proportion of dispatched urgency levels (with the highest urgency level as the primary criterion), years of employment, and the average call duration. The data used for matching covered a 3-month period from January 1, 2022, to March 31, 2022, both prior to the introduction of video streaming and preceding the pilot phase of the study.

Newly employed personnel where matching was not possible were randomly assigned to one of the two clusters. Before the study period, the implementation of video streaming in the intervention group occurred progressively over a span of 7 months (from June 1, 2022, to December 31, 2022), following a pilot phase that took place from April 19, 2022, to May 31.

2.4 Endpoints

Primary Outcome Measure

- The frequency of dispatches with the highest level of urgency (A-responses)

Secondary Outcome Measures

- The frequency of dispatches with the levels of urgency A, B, C, D and E,
- Mortality within 30 days after study inclusion,
- Number of participants where the levels of urgency are identical when comparing the ambulance to and from the scene,

- Length of stay at hospital,
- The number of participants needing an ICU admission at hospital,
- Number of emergency calls where the dispatched level of urgency is changed during the call,
- Number of emergency calls where the initially dispatched level of urgency is subsequently lowered,
- Number of emergency calls where the initially dispatched level of urgency is subsequently increased,
- Number of emergency calls where the allocated resources is changed during the call,
- Number of participants readmitted to hospital within 24 hours after a dispatch without hospital admission (lowest level of urgency - response level E),
- Duration of emergency medical calls,
- Time from emergency call to dispatch,
- On-scene time

Other Pre-specified Outcome Measures:

- Number of participants with return-of-spontaneous circulation (ROSC) after cardiac arrest
- 90 days neurological outcome after cardiac arrest measured by the cerebral performance scale (CPC)
- 90 days functional outcome after cardiac arrest measured by the modified Rankin Scale (mRS)
- Number of patients with Stroke or TIA identified by the EMS dispatcher in the EMDC
- Rate of revascularization treatment (intravenous thrombolysis and/or endovascular treatment (EVT)) among patients with acute ischemic stroke
- Primary admission to a hospital offering acute stroke treatment
- Onset-to-treatment times for patients with acute ischemic stroke treated with i.v. thrombolysis and onset-to-groin-puncture for patients treated with EVT.
- Number of lowest level of urgency (response level E) dispatched among children (< 15 years of age)

- Re-admissions among children (< 15 years of age) to hospital within 24 hours from a prior emergency call dispatched with lowest level of urgency (response level E)
- Number of dispatches for each level of urgency (response level A, B, C, D and E) among children (< 15 years of age)

3 Study Methods

3.1 General Study Design and Plan

Study type: Single center, prospective, cluster-randomized, unblinded trial.

Type of Comparison: superiority

Type of control: Matched control group (matched-pair design)

Randomization: Emergency calls received in the Emergency Medical Dispatch Center are allocated randomly between the intervention group and the control group. Given the extended call duration within the intervention group, it is anticipated that the control group (telephone-only) will experience a higher volume of calls.

3.2 Inclusion-Exclusion Criteria and General Study Population

All emergency calls received at the Emergency Medical Dispatch Center and handled by either the intervention or control group will be included in the study.

Inclusion criteria

- Emergency calls (1-1-2 calls equivalent to 9-1-1 calls),
- Received by an EMS dispatcher included in the study (intervention or control group/cluster).

Exclusion criteria

- All calls to the EMDC other than emergency calls (including those from hospitals and general practitioners, including those outside of regular hours),
- Emergency calls managed by EMS dispatchers who are newly employed within the study period, on long-term leave, or not employed until the end of the study period are excluded from the study,

- Emergency calls managed by technical dispatchers (logistical dispatcher restricted to dispatching only the highest urgency level).

3.3 Randomization

Emergency calls will be allocated at random to either of the two clusters: the intervention group utilizing video, or the control group utilizing telephone-only communication. Alternatively, some emergency calls will be directed to EMS dispatchers who are not part of the study.

3.4 Sample Size

The CAM-VISION trial is conducted using a convenience sample, with an anticipated count of emergency calls during the study period ranging between 18,000 and 20,000.

3.5 Timing of Analyses

The study period and its corresponding follow-up periods, as determined by specific variables, are finalized during the drafting of this SAP (Statistical Analysis Plan).

As of this SAP, data extraction has not been initiated due to pending data authorization. The subsequent actions, in chronological order, prior to data extraction and analysis, are outlined below:

SAP Signature

- By the principal investigator and the senior statistician.

Data Authorization

- The Regional Research Council (The Legal Office, Central Denmark Region) and
- The Danish Data Protection Agency.

Primary Analyses

- Primary and Secondary Outcomes

Secondary analyses

- Other Outcomes

3.6 Analysis Populations

Intention to Treat (video stream)

The intention-to-treat populations (intension-to-video-stream) includes all patients allocated to either exposure group. Binary outcomes are investigated using risk differences (RD) implementing linear regression and relative risks (RR) by Poisson regression. Robust variance estimation is applied in both regression analyses. Continuous outcomes are investigated using linear and Tobit regression, where the latter is implemented for time variables that are right censored. Time-to-event variables are

investigated using Cox-regression analysis, while CPC and mRS are investigated using ordered logistic regression and by the concordance index. To account for cluster randomization, we will implement cluster robust variance in all regression analyses with a separate cluster id for each EMS dispatcher. Effect measures are presented with 95% confidence intervals and all analyses are conducted in Stata 17.

No per-protocol analyses will be conducted because of substantial risk of selection bias.

3.7 Missing Data

The anticipated connection between treatment allocation and loss to follow-up is expected to be minimal. Thus, missing data, including outcomes, is assumed to be missing at random and will be handled using multiple imputation.
The extent of missing data will be presented for outcome variables to quantify the degree of missing data.

3.8 Summary of Study Data

- Summary tables will be structured (columns for each cluster)
- Descriptive or summary statistics that will be displayed for continuous, categorical and binary data.

All continuous variables will be summarized as mean with standard deviation or median with interquartile range (25th and 75th percentiles) calculated based on the non-missing data. For categorical and binary measures, the frequency and percentages (calculated based on the non-missing sample size) will be provided.
