# Supplementary material for: Video Streaming or Telephone Communication During Emergency Medical Services Dispatch Calls: A Cluster Randomized Clinical Trial
Source: JAMA Netw Open. 2025 Jul 1;8(7):e2519020. doi: 10.1001/jamanetworkopen.2025.19020 (PMC12215568; doi:10.1001/jamanetworkopen.2025.19020)
Supplement: Supplement 3. — Data Sharing Statement [file jamanetwopen-e2519020-s003.pdf]

Data Sharing Statement

Gude. Video Streaming or Telephone Communication During Emergency Medical Services Dispatch Calls. *JAMA Netw Open*. Published July 03, 2025. doi:10.1001/jamanetworkopen.2025.19020

Data
Additional Information: ClinicalTrials.gov Identifier: NCT05742412
Data available: Yes
Data types: Deidentified participant data
How to access data: The corresponding author, Martin F. Gude, at gude@dadlnet.dk.
When available: With publication

Supporting Documents
Document types: Statistical/analytic code
How to access documents: The corresponding author Martin F. Gude, at gude@dadlnet.dk
When available: With publication

Additional Information
Who can access the data: Deidentified participant data will be made available upon reasonable request and in compliance with Danish legislation. Requests for access to the data should be directed to the corresponding author, Martin F. Gude, at gude@dadlnet.dk.
Types of analyses: The data will be made available for analyses aimed at advancing scientific knowledge and improving emergency medical services. Specifically, this includes but is not limited to replication of the study findings, secondary analyses, and meta-analyses. Data access requests must align with the original ethical approvals and comply with Danish legislation.
Mechanisms of data availability: Data will be made available after approval of a research proposal and the execution of a signed data access agreement. Requests will be evaluated to ensure alignment with the original ethical approvals and compliance with Danish legislation. Investigator support will be provided as needed to facilitate appropriate use of the data.
Any additional restrictions: The use of the data is restricted to non-commercial research purposes. Data must be handled in compliance with Danish data protection laws and cannot be re-shared or used to identify individuals. Additionally, any publications resulting from the use of the data must acknowledge the original study and its investigators.
